# Characterization of serum miRNAs as molecular biomarkers for acute Stanford type A aortic dissection diagnosis

**DOI:** 10.1038/s41598-017-13696-3

**Published:** 2017-10-20

**Authors:** Zhenjun Xu, Qiang Wang, Jun Pan, Xia Sheng, Dongxia Hou, Hoshun Chong, Zhe Wei, Shasha Zheng, Yunxing Xue, Qing Zhou, Hailong Cao, Chen-Yu Zhang, Dongjin Wang, Xiaohong Jiang

**Affiliations:** 1 State Key Laboratory of Pharmaceutical Biotechnology, Jiangsu Engineering Research Center for MicroRNA Biology and Biotechnology, NJU Advanced Institute for Life Sciences (NAILS), School of life sciences, Nanjing University, 163 Xianlin Road, Nanjing, 210046 China; 2 Department of Thoracic and Cardiovascular Surgery, the Affiliated Drum Tower Hospital of Nanjing University Medical School, 321 Zhongshan Road, Nanjing, 210008 China; 3 Genscript Biotech Corporation, Genscript 860 Centennial Ave, Piscataway, NJ 08854 USA

## Abstract

Early and convenient diagnosis is urgently needed for acute Stanford type A aortic dissection (AAAD) patients due to its high mortality within the first 48 hours. Circulating microRNAs (miRNAs) are promising biomarkers of cardiovascular diseases, however, little is known about circulating miRNAs involved in AAAD. Here, the blood serum was sampled from 104 AAAD+ patients and 103 age-matched donors. Initial screening was conducted using the TaqMan Low Density Array followed by RT-qPCR confirmation. According to the two-phase selection and validation process, we found that miR-25, miR-29a and miR-155 were significantly elevated, while miR-26b was markedly decreased in AAAD+ serum samples compared with AAAD− individuals. Most importantly, for individuals with hypertension, which is a major contributor to AAAD, the 4-miRNA panel also showed high accuracy in predicting those who are more likely to develop AAAD. In the blind trial set, the panel correctly classified 93.33% AAAD+ patients and 86.67% controls from the hypertension cohort. Finally, the serum miRNA-based biomarker for early AAAD detection was supported by a retrospective analysis. Taken together, we identify a distinct profile of 4-miRNA that can serve as a noninvasive biomarker for AAAD diagnosis, especially for those with hypertension.

## Introduction

Aortic dissection (AD) is a catastrophe of the aorta, which is associated with high mortality^1–3^. AD occurs when a tear in the intimal layer of the aorta, causes abnormal blood flow within the layers of the aortic wall, forcing separation of the layers^2,3^. With an incidence of 3–5 cases per 100,000 people per year in general population, AD most frequently occurs in men in their 60 s and 70 s which incidence increased to 35 case per 100,000 people-years^3–5^. When left untreated, about 24% of patients die within the first 24 hours, 50% die within 48 hours^6,7^. In patients with undiagnosed ascending AD, the 3-month mortality rate approaches 90%^2,6,7^. The acute Stanford type A aortic dissection (AAAD), which occurs within two weeks from the onset of disease and involves in the ascending aorta and arch, accounts for about 67.3% of AD and always need for an emergency surgical treatment^2,8^. Given that the immediate mortality rate of AAAD increases 1% per hour during the first 48 hours, when an AAAD is detected early and treated promptly, the chance of survival greatly improves^3,6,7^. However, the symptoms of AAAD may mimic those of other diseases, such as acute coronary syndrome, pulmonary embolism or pneumothorax, often leading to delays in diagnosis^3,8,9^. Therefore, making early diagnosis and timely treatment are critical for patients to survive^3,10^. Nowadays, computed tomography (CT) and magnetic resonance imaging (NMR) are the most common initial and standard diagnostic tests performed in AAAD patients due to their high sensitivity^2,11^. Hypertension is an important contributor of AAAD, at least 75% of individuals with AAAD have a history of hypertension^4,12^. Notably, even with surgical treatment, the perioperative mortality of AAAD is remaining high (13~17%), indicating that prevent the onset of AAAD is particularly important to reduce the mortality of this devastating disease^13^. The noninvasive blood-based test is very attractive. Although blood D-dimer, elastin fragments, smooth muscle myosin heavy chain, matrix metalloproteinase are helpful in imaging evaluation, currently, there is no available routine blood test yet^13^. Thus, the identification of novel blood biomarkers that predict which subset of individuals is more likely to develop AAAD is particularly important for people with hypertension^10^.

MicroRNAs (miRNAs) are endogenous small, non-coding RNAs that posttranscriptionally regulate gene expression^14,15^. Emerging evidence showed that human plasma/serum contains numerous stable circulating miRNAs, either by passive leakage from broken cells or by active secretion via cell-derived extracellular vesicles^16,17^. As miRNAs are critical regulators of numerous disease process, the circulating miRNA are also differently expressed under pathological conditions, including cardiovascular diseases^18^. Several miRNAs have been reported to contribute to the formation and dilation of aortic aneurysm. For example, overexpression of miR-29 and miR-195 accelerated the degradation of extracellular matrix, and downregulation of miR-21, miR-143/145 promoted normal contractile and quiescent vascular smooth muscle cells (VSMCs) to synthetic and proliferating phenotype, leading to the degeneration of medial layer and formation of aortic aneurysm^19–22^. Circulating miRNAs are also have been proven to be useful biomarkers to monitor the dilation of the aneurysm^23,24^. Both aortic aneurysm and AAAD are severe vascular diseases and life-threatening^1,2^. Currently, the most studied miRNAs in aneurysm is the miR-29 family. Boon RA *et al*. summarized that TGF-β upregulated miR-29 expression, which directly targeting collagens and elastin, and consequently results in the formation and development of aneurysm^19^. Inhibition of miR-29, especially the entire miR-29 family reduced aneurysm formation, on the contrary, overexpression of miR-29b induced severe aneurysm expansion in different murine models^19,25,26^. In addition, several studies have reported that circulating miRNA expression patterns differ between aortic aneurysm patients and healthy controls, suggesting the diagnostic and therapeutic potential of miRNAs in this disease^24,25,27–29^. However, only a few studies have mentioned the different miRNA expression profiles in AD tissues, and the patient number is relatively small^30–32^. Moreover, as we known that hypertension is the most important contributor of AAAD, it is necessary to know whether we can use serum miRNA as biomarkers to predict which subset of individuals trends to develop AAAD^13^.

To investigate whether the serum miRNA expression panel can distinguish AAAD patients from healthy controls, or individuals with hypertension, we used TaqMan Low Density Array (TLDA) screening followed by an individual quantitative RT-PCR confirmation to systematically evaluate serum miRNA expression. In the present study, we obtained a profile of four serum miRNAs, which can serve as novel noninvasive biomarkers for early AAAD diagnosis.

## Results

### Characteristics of patients

**Figure 1:**
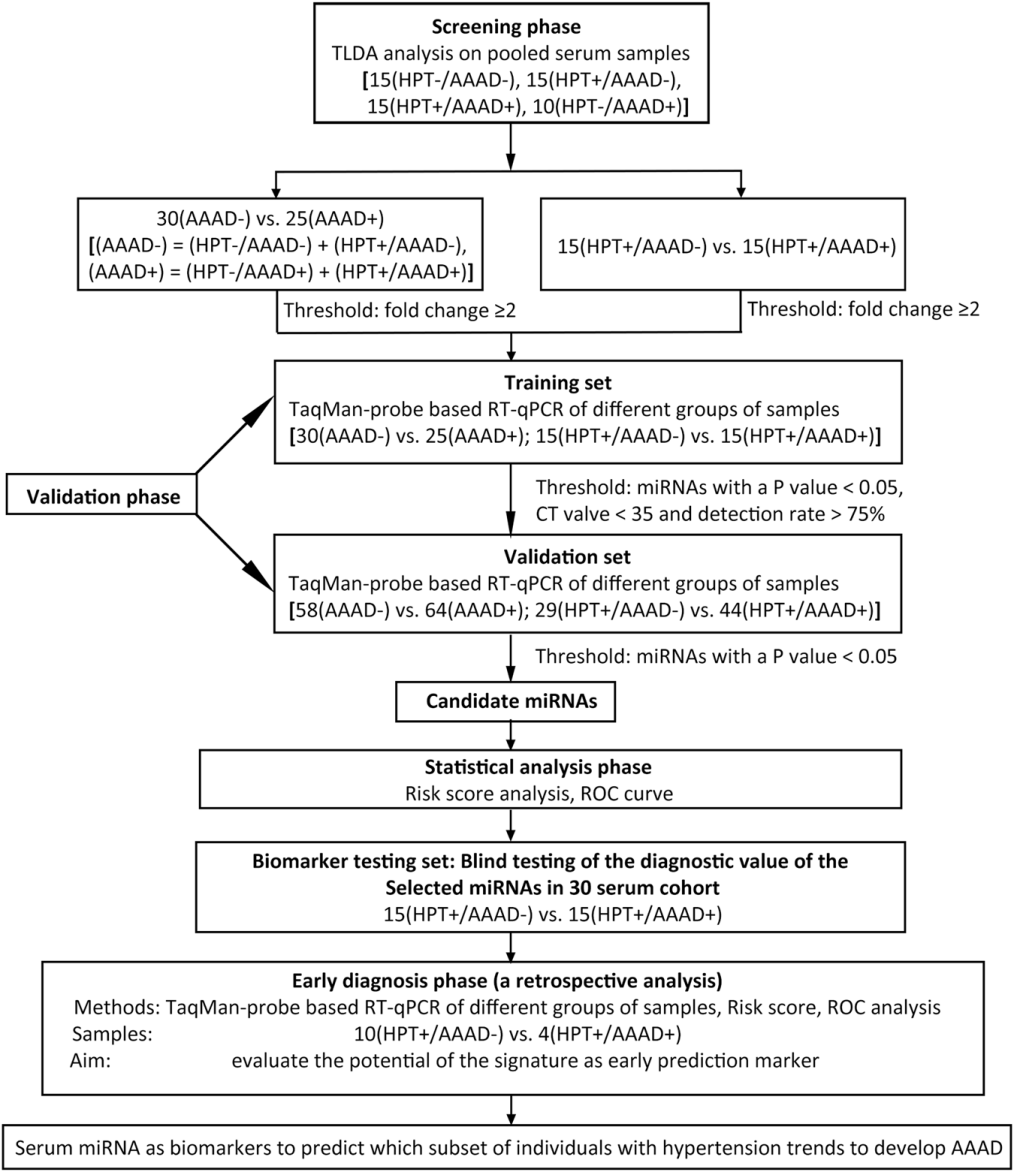
A flow chart of the study design strategy of patients with AAAD including training, validation and testing sets. HPT: hypertension; AAAD: acute Stanford A aortic dissection.

A total of 207 participants, including 104 AAAD+ patients (of which 74 with hypertension and 30 without hypertension), and 103 age-matched AAAD− individuals (of which 59 with hypertension and 44 without hypertension) were enrolled in this study. Table 1 shows the demographics and clinical features of different cohorts of people. As shown in Fig. 1, we designed a multiphase study to identify serum miRNAs as surrogate markers for predicting the prognosis of AAAD, especially for those individuals with hypertension. In the initial biomarker selection stage, serum samples were collected from Nanjing Drum Tower Hospital, Jiangsu, China. 25 AAAD+ cases, 30 matched AAAD− controls were subjected to ABI TaqMan Low Density array (TLDA) and RT-qPCR analysis. The significantly altered miRNAs were selected and validated in additional 64 AAAD+ cases and 58 AAAD− controls. Next, the predictive value of identified miRNA profile was evaluated in another cohort by blinded trail. Finally, the refined panel of serum miRNAs was tested in the prediagnosis serum samples to determine whether this biomarker set might be useful for early AAAD detection. There was no significant difference in the distribution of age between different groups of cohorts, while the AAAD+ group had more hyperlipidemia patients and alcoholic abusers than the AAAD− control groups. Neither the AAAD+ group nor AAAD− had any history of atherosclerosis, bicuspid aortic valve and cocaine abuse. All 3 patients with a history of aortic aneurysm and 5 Marfan’s syndrome patients were in normal blood pressure AAAD+ cohort (Table 1). In general, AAAD+ patients and AAAD− control subjects had no other significant cardiac dysfunction, active infection (hepatitis, tuberculosis, etc.), and neurological or psychiatric disorders at the time when blood was drawn.

**Table 1:** Demographic and clinical features of AAAD+ patients and AAAD− individuals.

	Training set(n = 55)			Validation set(n = 122)			P-value^3^ (AAAD+ in Training vs. validation
	AAAD+ (n = 25)	AAAD− (n = 30)	P-value^1^ (AAAD+ vs. AAAD−)	AAAD+ (n = 64)	AAAD− (n = 58)	P-value^2^ (AAAD+ vs. AAAD−)	
Age (years)	51.20 ± 12.50	51.47 ± 6.45	0.919^a^	51.25 ± 12.52	51.19 ± 6.16	0.974^a^	0.987^a^
Female	13	10	0.162^b^	18	20	0.134^b^	0.342^b^
Smoker	2	0	0.202^b^	9	0	0.003^b^	0.721^b^
Alcohol abuse	1	0	0.455^b^	6	0	0.029^b^	0.668^b^
Hypertension	15	15	0.458^b^	44	29	0.035^b^	0.433^b^
Hyperlipidemia	15	5	0.001^b^	38	10	<0.001^b^	0.957^b^
Atherosclerosis	0	0	—	0	0	—	—
Aortic aneurysm	1	0	0.455^b^	2	0	0.175^b^	0.837^b^
Marfan’s syndrome	1	0	0.455^b^	4	0	0.053^b^	0.679^b^
Bicuspid aortic valve	0	0	—	0	0	—	—
Cocaine abuse	0	0	—	0	0	—	—
Diabetes	0	0	—	9	1	0.018^b^	0.057^b^
Previous AD	2	0	0.202^b^	6	0	0.029^b^	0.838^b^

AAAD: acute Stanford type A aortic dissection; AD: Aortic dissection.

Statistical comparison was performed by using Student’s t-test (a) or two-sided λ^2^ test (b).

### Screening of serum miRNAs of AAAD by TLDA analysis

**Figure 2:**
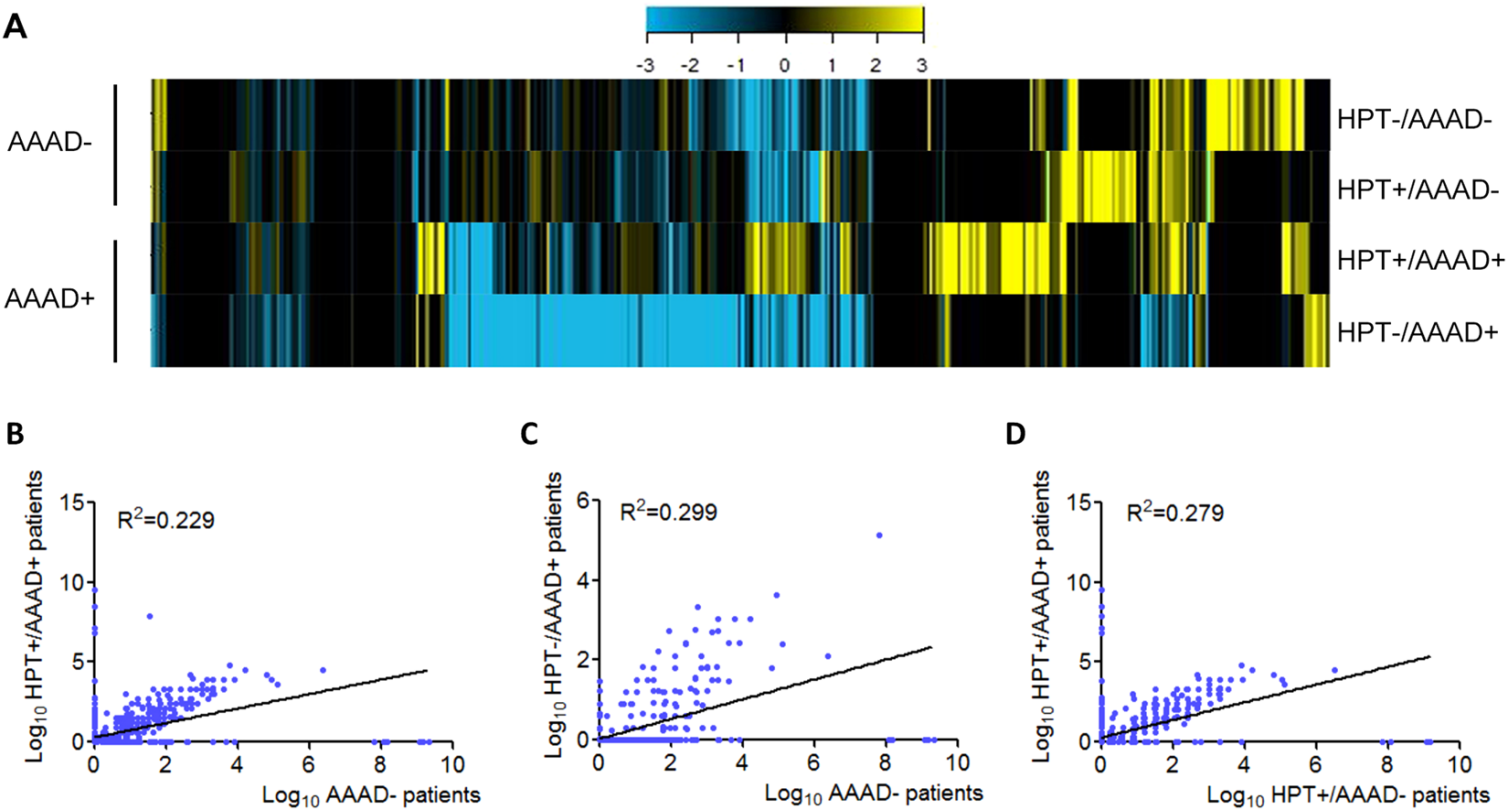
MiRNA profiles in AAAD+ and AAAD− groups detected by TLDA. (**A**) Heat map showing the relative expression of miRNAs that changed in the serum of AAAD− patients (HPT−/AAAD− and HPT+/AAAD−) and AAAD+ individuals (HPT+/AAAD+ and HPT−/AAAD+). Each sample comprised a pool of serum samples of indicated group. Each column depicts an individual miRNA. The fold change for the samples is colour-coded according to the key. (**B**–**D**) Pearson’s correlation scatter plot of plasm miRNA levels between AAAD+ and AAAD− groups; (**B**) between HPT+/AAAD+ patients and AAAD− groups; (**C**) between HPT−/AAAD+ patients and AAAD− groups; (**D**) between HPT+/AAAD+ patients and HPT+/AAAD− individuals. HPT: hypertension; AAAD: acute Stanford A aortic dissection.

To screen candidate serum miRNAs for AAAD detection, we used the TLDA analysis. The expression of miRNAs in the serum was screened to identify significantly altered miRNAs between AAAD+ patients and AAAD− controls. Considering the primary role of hypertension in AAAD, total RNAs were extracted from serum samples of AAAD− controls with normal blood pressure (HPT−/AAAD−), AAAD− individuals with hypertension (HPT+/AAAD−), AAAD+ patients with normal blood pressure (HPT−/AAAD+), and AAAD+ patients with hypertension (HPT+/AAAD+), respectively. Equal concentrations of total RNAs were analyzed using TLDA microarray. Of the 760 miRNAs the array chip, 313 serum miRNAs were detected in either healthy individuals or the other three groups of patients (Supplementary Table S1). As shown in Fig. 2A, different miRNA profiles were observed in AAAD− and AAAD+ groups of serum samples, suggesting the potential of using serum miRNAs as biomarkers. Of note, according to the heat map, the serum miRNAs were significantly different expressed in AAAD+ patients with or without hypertension (HPT+/AAAD+ and HPT−/AAAD+). This is reasonable because some AAAD+ patients with normal blood pressure have Marfan’s syndrome, aortic aneurysm or other undiagnosed pathogenesis, which were completely different pathologies from hypertension-associated AAAD. Therefore, we further investigate whether miRNA profiles can directly separate HPT−/AAAD+ and HPT+/AAAD+ patients from AAAD− individuals, respectively. By using Pearson’s correlation scatter plots, we compared the miRNA expression profiles between HPT+/AAAD+ and AAAD− group. The square of the Pearson’s correlation coefficient (R^2^) value was only 0.229 between the HPT+/AAAD+ and AAAD− group, indicating complete different miRNA profiles between the two group of sample (Fig. 2B). Between HPT−/AAAD+ and AAAD− group, the R^2^ value was 0.299, also showed complete different miRNA profiles between the two groups of samples (Fig. 2C). Moreover, because hypertension contributes to at least 75% of AAAD, it is important to know whether serum miRNAs can be used as biomarkers to predict which subset of individuals with hypertension trends to develop AAAD. As shown in Fig. 2D, the R^2^ value of the miRNA expressions between HPT+/AAAD− and HPT+/AAAD+ group was 0.279, suggesting that serum miRNAs are promising prognostic biomarkers for HPT+/AAAD+ patients. 158 serum miRNAs were found to be differentially expressed between the HPT+/AAAD+ and HPT+/AAAD− groups, of which 82 were up-regulated and 76 were down-regulated (Supplementary Table S2). In this phase, we selected miRNAs that qualify the following criteria for additional RT-qPCR validation: 1) miRNAs with a mean fold change ≥2 between the HPT+/AAAD+ and AAAD− were selected; 2) miRNAs with a mean fold change ≥2 between the HPT−/AAAD+ and AAAD− were selected; 3) miRNAs with a mean fold change ≥2 between the HPT+/AAAD− and HPT+/AAAD+ were selected; 4) miRNAs change in the same direction (all went up or down) in three compared set. Using these criteria, 22 miRNAs were identified and chosen for further analysis (Supplementary Table S3).

### Validation of candidate serum miRNAs by RT-qPCR

**Figure 3:**
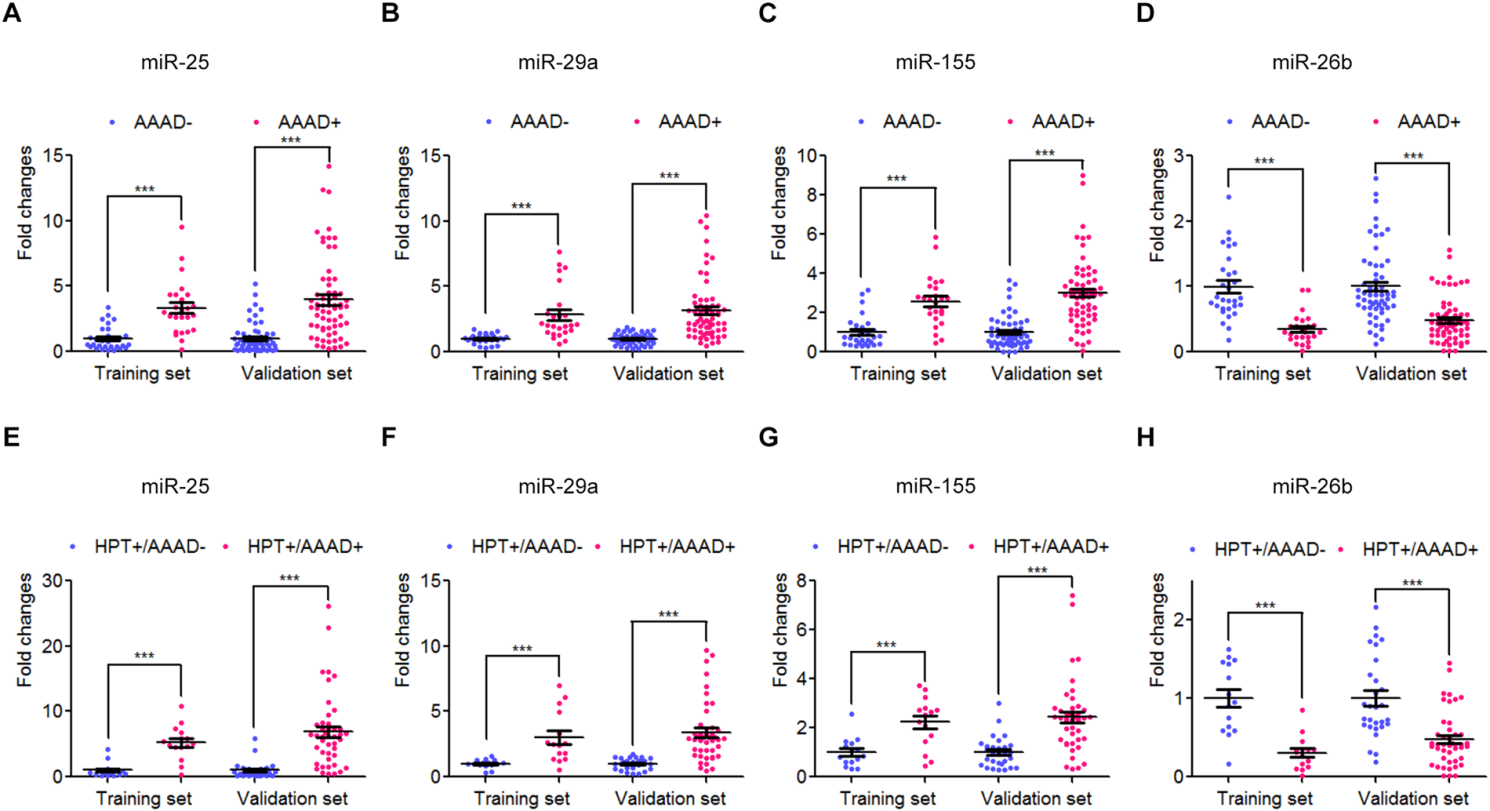
Differential expressions of the four selected serum miRNA between AAAD+ patients and AAAD− controls, and between HPT+/AAAD+ patients and HPT+/AAAD− individuals in training set and validation. (**A**–**D**) Fold changes between AAAD+ patients and AAAD− controls, miR-25 (**A**), miR-29a (**B**), miR-155 (**C**), miR-26b (**D**); (**E**–**H**) Fold changes between HPT+/AAAD+ patients and HPT+/AAAD− individuals, miR-25 (**E**), miR-29a (**F**), miR-155 (**G**), miR-26b (**H**). HPT: hypertension; AAAD: acute Stanford A aortic dissection. *P < 0.05; **P < 0.01; ***P < 0.001.

The differential expression of these 22 selected miRNAs was confirmed in two independent cohorts of 89 AAAD+ patients (59 (HPT+/AAAD+) and 30 (HPT−/AAAD+)) and 88 AAAD− control individuals (44 (HPT−/AAAD−) and 44 (HPT+/AAAD−)) using RT-qPCR analysis. In this phase, we first validated the differential expression of these 22 selected miRNAs in 30 AAAD− and 25 AAAD+ individual serum samples using RT-qPCR analysis in the training set. Differentially expressed miRNAs (P < 0.05) were chosen only when they showed a parallel trend in the variation among the four groups in both the screening and validation phases. The expression levels of miR-25, miR-29a and miR-155 were elevated, while miR-26b was markedly decreased in the serum samples from AAAD+ patients compared with AAAD− controls in the training set (Supplementary Table S4 and Fig. 3A–D). However, the expression levels of 18 selected miRNAs were either detection rate <75% or not significantly altered.

To verify the accuracy and specificity of these 4 miRNAs to be used as the AAAD+ signature, we further assessed the 4 miRNAs in a larger population sample set consisting of 64 AAAD+ patients (44 (HPT+/AAAD+) and 20 (HPT−/AAAD+)) and 58 AAAD− controls (29 (HPT−/AAAD−) and (29 (HPT+/AAAD−)). As shown in Fig. 3A–D, miR-25, miR-29a, miR-155 were up-regulated, whereas miR-26b was found to be down-regulated in AAAD+ patients compared with AAAD− control individuals. More importantly, the similar trend of miRNA expression alteration was observed in HPT+/AAAD+ group compared with HPT+/AAAD− individuals, suggesting these 4 miRNAs were also capable of distinguish the subset of AAAD+ patients from individuals with only hypertension (Fig. 3E–H).

Through this two-phase test and analysis, a panel of 4-miRNA (miR-25, miR-29a, miR-155 and miR-26b) was generated and have the potential to be novel biomarkers for AAAD, including HPT+/AAAD+ and HPT−/AAAD+ patients, in the next test and analysis.

### Prediction of AAAD cases and control subjects by risk score analysis

To further evaluate the diagnostic value of this 4-miRNA profiling system, we performed a risk score analysis on the data set and employed this risk scoring method to predict AAAD cases and control (AAAD−) subjects. First, the risk score formula was used to calculate the risk scores for all samples in the training set (25AAAD+ and 30AAAD−). Samples were ranked according to their risk scores and then divided into a high-risk group, representing the predicted AAAD+ cases, or a low-risk group, representing the predicted AAAD− subjects, using the optimal cutoff value of 46.50%. At this cutoff, the sensitivity was 96.00% and the specificity was 100.00%, with the value of sensitivity+ specificity considered to be maximal. As shown in Supplementary Table S5, none of the 30 AAAD− samples had a risk score >46.50%, while 24 out of the 25 AAAD+ samples had a risk score >46.50%. Second, the risk score formula, using the same cutoff point, was used to calculate the risk score for samples from the validation set. The sensitivity for the validation set was 89.06% and the specificity was 94.83%. Out of 64 AAAD+ cases and 58 AAAD− individuals from the validation set, only 3 AAAD− samples and 7 AAAD+ cases were incorrectly predicted by this risk score method.

**Figure 4:**
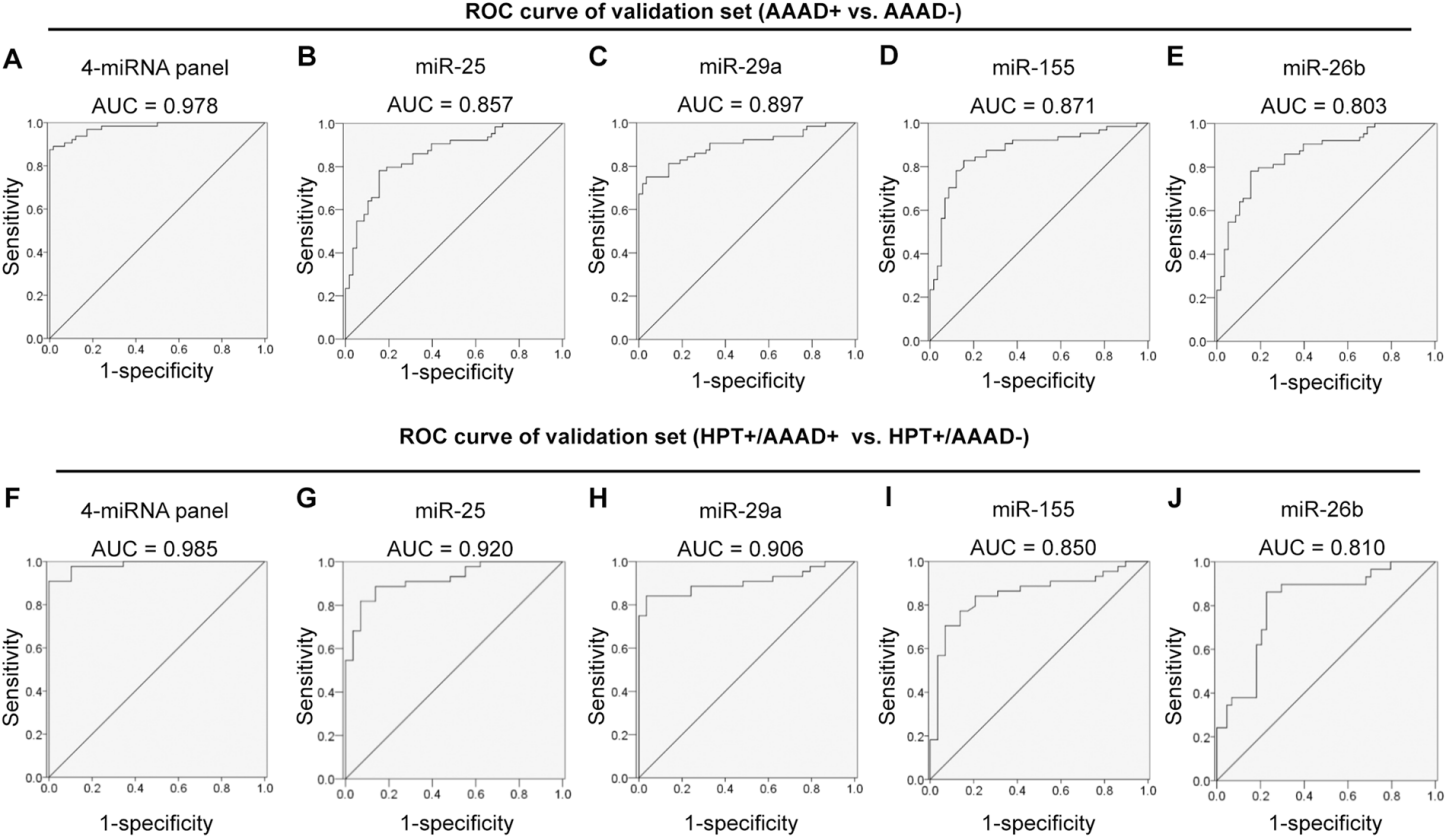
ROC curve analysis for discrimination between AAAD+ patients and AAAD− control serum samples and between HPT+/AAAD+ patients and HPT+/AAAD− control samples based on the three-serum profile. (**A–E**) ROC curve for the ability of miR-panel (**A**), miR-25 (**B**), miR-29a (**C**), miR-155 (**D**) and miR-26b (**E**) to separate AAAD+ patients from AAAD− individuals the in validation set; (**F–J**) ROC curve for the ability of miR-panel (**F**), miR-25 (**G**), miR-29a (**H**), miR-155 (**I**) and miR-26b (**J**) to separate HPT+/AAAD+ patients from HPT+/AAAD− individuals the in validation set. HPT: hypertension; AAAD: acute Stanford A aortic dissection.

We also constructed receiver operating characteristic (ROC) curves for continuous predictors using these risk score functions to estimate the sensitivity and specificity of the miRNA-based biomarkers. The areas under the curve (AUC) were 0.995 and 0.978 for the training set and validation set, respectively (Fig. 4A and Supplementary Table S5). The results indicate that the profile of the 4-serum miRNAs is an accurate biomarker for AAAD diagnosis. To illustrate the contribution of individual serum miRNAs on the AUC of the ROC curve, we established ROC curves to evaluate the diagnostic value of each miRNA for differentiating between AAAD+ cases and AAAD− cases. We found that the AUC for individual miRNAs (miR-25, miR-29a, miR-155 and miR-26b) can be 0.881, 0.899, 0.863, 0.911 in training set and 0.857,0.897, 0.871, 0.803 in validation set, respectively (Fig. 4B–E and Supplementary Table S5). These results clearly indicate that, although one particular miRNA in serum can help distinguish AAAD+ cases from AAAD− cases, a combination of a panel of miRNAs has a great potential to offer much more sensitive and specific diagnostic tests.

In the comparison between HPT+/AAAD+ and HPT+/AAAD− samples, these 4 miRNAs continued showing high predictive power. AUC of miR-25, miR-29a, miR-155 and miR-26b in predicting AAAD individually are 0.920, 0.906, 0.850 and 0.810 in validation set (Fig. 4G–J and Supplementary Table S5). We then predicted HPT+/AAAD+ patients in HPT+ individuals (including HPT+/AAAD+ and HPT+/AAAD−) using the 4-miRNA panel. With an optimal cutoff value of 36.89%, at which the sum of the sensitivity and specificity was maximal, the specificity was 82.76% and the sensitivity was 97.73%, and AUC of 0.985 in validation set (Fig. 4F and Supplementary Table S5). Combined with the previous result, it shows that these 4 miRNAs can be used to diagnose AAAD with high accuracy in both hypertension sub-populations and total population.

### Evaluation of the predictive value of the four-miRNA panel by blinded trial

**Figure 5:**
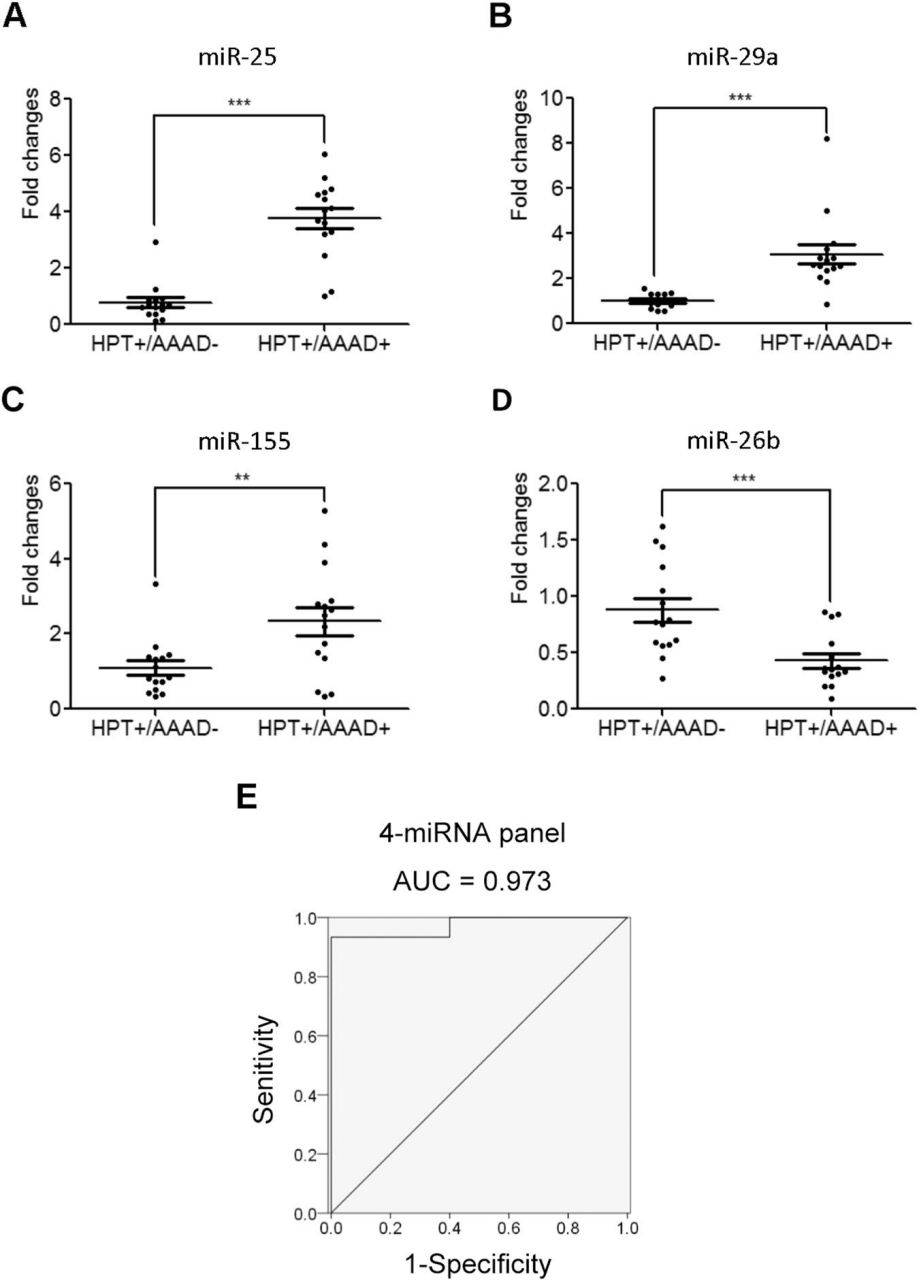
Differential expression of the four selected serum miRNA and the ROC curve analysis for the risk score between HPT+ AAAD+ patients and HPT+/AAAD− individuals in the blind trail set. (**A**–**D**) Fold changes between HPT+/AAAD+ patients and HPT+/AAAD− controls, miR-25(**A**), miR-29a (**B**), miR-155 (**C**), miR-26b (**D**); (**E**) ROC curve analysis for the ability of four-miRNA panel to separate the HPT+ AAAD+ patients from HPT+/AAAD− individuals in the blind trail set. HPT: hypertension; AAAD: acute Stanford A aortic dissection. *P < 0.05; **P < 0.01; ***P < 0.001.

We tested an additional 30 serum samples in a blind trial using the RT-qPCR assay, including 15 HPT+/AAAD+ cases and 15 HPT+/AAAD− controls (Supplementary Table S6). We aimed to further confirm the diagnostic accuracy of the four serum miRNA-based biomarker for differentiating AAAD+ patients from individuals with hypertension only. Next, we analyzed the relative levels of the four miRNAs from the 30 serum samples and classified them on the basis of the risk score model of the four-miRNA panel, with a cutoff value of 36.89%. As a result, 14 of 15 HPT+/AAAD+ samples (93.33%) and 13 of 15 HPT+/AAAD− samples (86.67%) were classified correctly (Table 2). As shown in Fig. 5A–D, the relative concentrations of miR-25, miR-29a and miR-155 were significantly higher in HPT+/AAAD+ patients than in HPT+/AAAD− control individuals, whereas miR-26b was markedly decreased (P < 0.05). In the blind cohort, the AUCs for the 4-miRNA ranged from 0.764 to 0.987 (Table 2). The four miRNA panel appeared to be reliable when applied to the testing set, exhibiting a sensitivity of 93.33%, a specificity of 86.67% and an AUC of 0.973 (Fig. 5E and Table 2). Taken together, the data suggest that the four-miRNA panel was accurate enough to distinguish HPT+/AAAD+ from HPT+/AAAD− patients.

**Table 2:** ROC curves of HPT+/AAAD+ patients and HPT+/AAAD− individuals in blinded trail set.

Risk score	AUC (95% CI)	P-value	Cutoff point	Sensitivity	Specificity	PV+	PV−
4-miR panel	0.973 (0.918–1.000)	<0.001	36.89%	93.33%	86.67%	87.50%	92.86%
miR-25	0.987 (0.937–1.000)	<0.001	1.353	86.67%	93.33%	92.86%	87.50%
miR-29a	0.951(0.856–1.000)	<0.001	1.354	93.33%	93.33%	93.33%	93.33%
miR-155	0.764 (0.576–0.952)	0.014	1.457	73.33%	86.67%	84.62%	76.47%
miR-26b	0.822(0.668–0.977)	0.003	0.500	73.33%	86.67%	84.62%	76.47%

ROC: receiver operating characteristic; HPT: hypertension; AAAD: acute Stanford type A aortic dissection; AUC, area under curves; CI, confidence interval; PV+, positive predictive value; PV−, negative predictive value.

### The 4-serum miRNA profile as serum biomarker for HPT+/AAAD+ prediction

We further evaluated the potential of using this 4-miRNA panel as biomarkers for AAAD prediction by using a retrospective analysis. In this analysis, serum samples were recruited from 20000 individuals who participated in a community-based screening program for noninfectious diseases conducted in the Jiangsu province during 2009 and 2010. Subsequently, individuals who had hypertension but had no history of AD were submitted to follow-up conducted in July of 2013. There were 4 individuals from this pool of 20000 who had been diagnosed as AAAD. The prediagnosis serum samples of these 4 AAAD cases, as well as 10 randomly selected AAAD−controls, were obtained and assessed by the RT-qPCR assay. As shown in Tables 3, 3 out of 4 subjects actually had a risk score >36.89% and were subsequently classified as AAAD+ cases by the 4-serum miRNA signature. Since these 3 subjects were “AD free” at recruitment but later clinically classified as AAAD, this result strongly suggests that our serum miRNA-based biomarker might be capable of predicting AAAD. These results significantly strengthen the clinical applicability of the 4-serum miRNA signature as an early serum biomarker of AAAD prediction.

**Table 3:** Risk score of the prediagnosis samples.

	Pre-1	Pre-2	Pre-3	Pre-4
Age (years old)	76	62	62	65
Sex	Female	Male	Male	Female
Smoking status	No	No	No	No
Alcoholic abuse	No	No	No	No
Hypertension	Yes	Yes	Yes	Yes
Hyperlipidemia	No	No	No	No
Atherosclerosis	No	No	No	No
Diabetes mellitus	No	No	No	No
Previous aortic dissection	No	No	No	No
Survival status	A	A	A	A
Leading time (year)	5	3	5	5
Risk score	51.26%	26.43%	39.39%	50.75%

Leading Time = Diagnostic time − blood drawing time; A = Alive after surgery.

## Discussion

About 25%–50% of AAAD patients have been misdiagnosed on the initial evaluation, because similar symptoms are always observed in myocardial infarction and other cardiovascular diseases patients. Early and convenient diagnosis is urgently needed for AAAD due to its high mortality within the first 48 hours. Emerging evidence suggests that the unique patterns of serum miRNA may serve as novel noninvasive biomarkers for various diseases, including cardiovascular diseases. In the present study, we identified a new four-miRNA panel which can serve as a potential biomarker for diagnosing AAAD patients. More importantly, for individuals with hypertension, the panel of miRNA might be useful to predict those who are more likely to develop AAAD.

Because it is not easy to diagnose AAAD by initial evaluation, once it bust, the patients can die of major bleeding within several hours. Thus, rather than curing this disease, it is more realistic to find a way to diagnose it in advance. There are several advantages for using serum miRNA in clinical diagnosis or screen of AAAD: (1) unlike transesophageal echocardiography, the serum-based biomarker is non-invasive; (2) unlike traditional CT or NMR, the serum-based biomarker is time-saving, low cost, and available at bedside; (3) The combination of these four miRNAs forms a more complete indicator for AAAD detection than the conventional single protein based biomarkers. As shown in Fig. 4 and Supplementary Table S5, AUC, sensitivity, and specificity for AAAD detection by our 4-miRNA biomarker are 0.995, 96% and 100%, respectively. These are significantly higher than those of any single-factor index, such as D-dimer, which has been widely used in routine clinical practice (specificity ~0.56).

To bring circulating miRNAs into routine diagnosis, reproducibly validated studies are indispensable. For overall population, the proportion of AAAD patients is relatively low. Thus, even AAAD can be predicted in total population with as high as 96% accuracy, there are still certain false positive cases exist. As we known, hypertension has become the most important risk factor for cardiovascular and cerebrovascular diseases worldwide, including AAAD^33^. According to the 2011 American College of Cardiology Foundation/American Heart Association hypertension guidelines (≥140/90 mm Hg), approximately 30% of adults in the United States and 27% of Chinese have hypertension^33,34^. In the present study, we first selected the candidate serum miRNAs, then validated the applicability of this miRNA-based biomarker in another independent hypertension cohort in a blind fashion. We found that three serum miRNAs, including miR-25, miR-29a and miR-155, were significantly elevated in HPT+/AAAD+ patients compared with HPT+/AAAD− group, whereas miR-26b was markedly decreased. The AUC of ROC curve of this 4-miRNA panel was 0.985 in the validation set cohort. Furthermore, in a single-blind sensitivity trial, using a proper cutoff value of 36.89%, the miRNA panel correctly classified 14 of 15 (93.33%) AAAD+ cases and 13 of 15 (86.67%) hypertension controls. Our results confirmed the effectiveness of this four-miRNA panel has high sensitivity and specificity for diagnosing of HPT+/AAAD+ patients from hypertension group.

Several studies have reported the differential expression of miRNAs in aortic tissue in patients with AAAD. However, only one study, with sample size of 20 AAAD patients and 20 controls without cardiovascular disease, have assessed the upregulated expressed circulating miRNAs by microarray analysis, including miR-4313, miR-933, miR-1281 and miR-1238^31^. In addition, few studies have profiled the miRNA expressions by using AAAD patients’ tissues. By comparing 6 patients with 6 controls, Liao *et al*. reported that miR-553, miR-183–3p, miR-419-3p and miR-338-5p and were upregulated, while miR-143, miR-145, miR-22 and miR-26b was significantly downregulated in the aortic tissue of AAAD patients, in which the change of miR-26b was consistent with our study^30^. Kimura *et al*. also profiled miRNA expression using aortic tissues from 8 AAAD patients and 9 controls, they showed that 33 miRNAs was upregulated and 8 miRNAs was significantly downregulated (>2-fold). However, only the upregulation of miR-21-5p was validated by RT-PCR^35^. Moreover, aberrant upregulated miR-30a and decreased miR-124 was also observed in the aortic tissue of AAAD patients^36,37^. As mentioned above, even in aortic tissue detection, the miRNAs identified by different groups vary from one another. This inconsistency, maybe because of the differences in patient races, sample size, methodology or the method of data normalization. Notably, in our study, the fold changes of the miRNAs in TLDA were not matched well with RT-qPCR analysis, which might be due to the 12-cycle pre-amplification that was made in TLDA. Therefore, the microarray results must be validated using RT-qPCR assay. In addition, appropriate normalization is critical for accurate RT-qPCR quantification, however, one common problem in circulating miRNAs studies is that lack of consensus endogenous controls. Several different genes, such as RNU44, RNU6B and miR-16 have been used as internal controls in previous studies. In our preliminary studies, the possible internal control hsa-miR-16 was tested for its reliability as internal controls. As shown in Supplementary Figure S1A, the expressions of miR-16 were not consistent between each group, thus ruling out its potential as stable internal control. In addition, we introduced synthetic cel-miR-39 and MIRNA2911, corresponding to *C. elegance* and honeysuckle MIRNA (MIR-2911), chosen because of the absence of homologous sequences in humans, into human serum after the addition of a denaturing solution that inhibits RNase activity. Notably no overt modulation of expression levels of cel-miR-39 or MIR2911 were observed (Figure S1B and C). Therefore, MIRNA2911 was chosen as suitable external standards. The use of this external control MIRNA for normalization improves the reproducibility and sensitivity of the results.

The biological relevance in cardiovascular diseases of the selected miRNAs identified in our study have been investigated in previous studies. MiR-25 was up-regulated in heart failure, both in mice and humans, which contributes to declining cardiac function during heart failure, and injection of miR-25 antagomiR markedly halted established heart failure in a mouse model, improving cardiac function^38^. Circulating miR-25 was also identified as an independent factor for intracranial aneurysms occurrence^39^. MiR-29 family target at least 16 genes which are involved in ECM remodeling in several organs, such as collagen isoforms, fibrillin-1 and elastin^40,41^. Recently, miR-29 has been indicated to play a pivotal role in the formation of aneurysm, systemic anti-miR-29 treatment decreased aortic dilatation in aged Ang II treated mice^25^. MiR-155 is ubiquitously expressed, it represents a typical multi-functional miRNA that regulates multiple biological pathways^42^. Emerging evidence suggests a significant role for miR-155 in immunity and inflammation, it has been related to several vital inflammatory processes in atherosclerosis^43^. MiR-26b has been reported to play a protective role in heart hypertrophy, overexpression of miR-26b in the heart inhibited up-regulation of its targets and the development of hypertrophy^44^. Because VSMCs play a critical role in the degradation of middle layer in AAAD^45^, we therefore discussed the possible associations of these 4 miRNAs and SMC functions. It has been reported that in VSMC differentiation models, overexpression of miR-29a promoted VSMC differention by targeting YY1^46^. In addition, miR-155 was upregulated in atherosclerosis, which resulted in VSMC proliferation and its directional migration from the middle layer into the neointima^47,48^. Moreover, miR-26b was decreased in monocrotaline-induced pulmonary artery hypertension rat model, which promoted proliferation of pulmonary arterial smooth muscle cells^49^. Taken together, the identified four-panel miRNA reflects various aspects of vascular functions, including collagen degeneration, inflammation, apoptosis, especially in VSMC dysregulation, thus the combination of these miRNAs forms a more complete indicator for AAAD detection than the single-miRNA or molecule based biomarker.

The potential of this 4-serum miRNA-based biomarker in the prediction of AAAD occurrence in hypertension cohort is intriguing. Although the number of tested AAAD cases was relatively small, the accuracy rate of this serum miRNA was inspiring. Three out of four pre-diagnosed serum samples were classified as AAAD, whereas none of the ten randomly picked hypertension control samples showed a risk score higher than the threshold. Because proper early treatment can be lifesaving for AAAD patients, our data strongly suggest that the 4-serum miRNA profile as biomarker for defining the early events of AAAD occurrence in hypertension people.

In summary, we have identified a distinctive serum miRNA panel in AAAD patients, which can serve as a noninvasive biomarker for AAAD detection, especially for those with hypertension. Our findings may provide additional information regarding the molecular mechanisms of aortic dissection. This serum miRNA-based biomarker may shed light on future clinical application.

## Materials and Methods

### Patients and controls

All samples were collected according to protocols approved by the Medical Ethics Committee from Nanjing University and Nanjing Drum Tower Hospital. Volunteers with or without hypertension and all patients signed informed consent for the collection and use of their samples for this study. All patients were diagnosed as AAAD by preoperative computed tomographic angiography and confirmed in surgery. The time from AAAD onset to surgery for all patients were less than 14 days. And all blood sample of AAAD+ group were collected within 48 hours from AAAD onset.

### Sample preparation

Blood samples were collected by syringe Blood samples were collected in PAXgene blood RNA tubes (Qiagen, Valencia, CA, USA) and then stored at −40 °C. The blood samples were transported on blue ice and stored at −80 °C.

#### ABI TLDA microarray analysis

The Megaplex TLDA, v2.0 (TaqMan® Low Density Array, Applied Biosystems (ABI), Foster City, CA) platform was used to measure miRNA expression. Briefly, the procedure begins with the retro-transcription of 70 ng of total RNA with stem-loop primers to obtain a cDNA template. A pre-amplification step was included in order to increase the concentration of the original material and to detect microRNAs that are expressed at low levels. The pre-amplified product was loaded into the TaqMan® Low Density Arrays and amplification signal detection was carried out using the 7900 FAST real time thermal cycler (ABI). After masking the adaptor sequences, the clean reads were aligned to the reference human (*Homo sa*piens) genome.

#### RNA isolation and RT-qPCR assays

Total RNA was extracted from 100 μL of blood sample using TRIzol Reagent (TaKaRa, Dalian, China). Briefly, 100 μL of blood sample was mixed with 1 mL of TRIzol Reagent (TaKaRa) in a 1.5 mL microcentrifuge tube (RNase-free, Axygen). The mixture was vortex-mixed vigorously for 10 s and then added to 5 μL of synthetic MIR2911 or Cel-miR-39. The mixture was further vortex-mixed vigorously for 20 s, and 200 μL of chloroform was then added. The mixture was vortex-mixed vigorously for 30 s, incubated on ice for 10 min, and then centrifuged at 16,000 g for 20 min at 4 °C. The supernatant was transferred to a new 1.5 mL microcentrifuge tube. Then, the same volume of isopropanol was added to the supernatant. And the tube was inverted 10 times to mix thoroughly. Next, the mixture was stored at −20 °C for 1.5 h and then centrifuged at 16,000 g for 20 min at 4 °C. Subsequently, the RNA pellet was collected, washed once with 75% ethanol and dried for 20 min at room temperature. Finally, the pellet was dissolved in 40 μL of RNase-free water and stored at −80 °C until further analysis.

The RT-qPCR assays were conducted on the LightCycler 480 Real-Time PCR system (Roche) according to the manufacturer’s instructions. Reverse transcriptions were performed as previously described with minor modification. In brief, 2 μL of total RNA was reverse-transcribed to cDNA using AMV reverse transcriptase (TaKaRa, Dalian, China) and a stem-loop RT primer (GenePharma, Shanghai, China). The reaction conditions were as follows: 16 °C for 30 min, 42 °C for 30 min and 85 °C for 5 min. Then, 2 μL of cDNA generated from reverse transcription was used for PCR analysis, and real-time PCR was performed using hydrolysis probes of piRNAs (custom-made probes from GenePharma) on a Roche LightCycler 480 PCR system. The reactions were incubated in a 96-well optical plate at 95 °C for 5 min, followed by 40 cycles of 95 °C for 15 s and 62 °C for 1 min. All reactions were run in triplicate. After the reaction, the CT values were determined using the fixed threshold settings, and the mean CT values were determined from triplicate PCRs.

#### Risk score analysis

Risk score analysis was performed to evaluate the association between aortic dissection and the miRNA expression levels in overall or hypertension populations. The risk score of each miRNA in the training set, denoted as “*S*”, was set as 1 if the expression level was lower than the lower 5% reference interval limit for the corresponding miRNA level in controls and as 0 if otherwise. Taking into account the association of each miRNA with infertility risk, each patient was assigned a risk score function (RSF) according to a linear combination of the miRNA expression levels. The RSF for sample *i* using the information from the miRNA level was:

$$rs{f}_{i}=\sum _{j=1}^{n}{W}_{j}\,\cdot \,{S}_{ij}$$

In the equation (1), *S*_*ij*_ is the risk score for miRNA *j* on sample *i*, and W_*j*_ is the weighting given to the risk score of miRNA *j*. To determine the *W·S*, *n* univariate logistic regression models were fitted using the disease status with each of the risk scores. The regression coefficient of each risk score was used as the weight to indicate the contribution of each miRNA to the RSF. Frequency tables and ROC curves were then used to evaluate the diagnostic effectiveness of the miRNA profile as infertility biomarkers and to find an appropriate cutoff point. Then the cutoff values determined in the training sample set were applied in the validation sample set.

### Statistical analysis

Statistical analysis was performed with SPSS 22.0 software. MicroRNA concentration data were represented as the means ± SEs. The t-test was used to compare differences of blood miRNA expression levels between aortic dissection groups and the control groups. Comparisons of *P*-value < 0.01 were considered statistically significant. The ROC curve was constructed to evaluate the prediction ability of the selected miRNAs for acute Stanford type A dissection aortic dissection in total population or in people with hypertension.

## Electronic supplementary material

Supplementary Info
