# Supplementary material for: Characterization of serum miRNAs as molecular biomarkers for acute Stanford type A aortic dissection diagnosis
Source: Sci Rep. 2017 Oct 20;7:13659. doi: 10.1038/s41598-017-13696-3 (PMC5651857; doi:10.1038/s41598-017-13696-3)
Supplement: Supplementary file 1 — Supplementary Info [file 41598_2017_13696_MOESM1_ESM.pdf]

Title: Characterization of serum miRNAs as molecular biomarkers for acute Stanford type A aortic dissection diagnosis

Zhenjun Xu[2†], Qiang Wang[2†], Jun Pan[2†], Xia Sheng[3], Dongxia Hou[1], Hoshun Chong[2], Zhe Wei[1], Shasha Zheng[1], Yunxing Xue[2], Qing Zhou[2], Hailong Cao[2], Chen-Yu Zhang[1*], Dongjin Wang[2*], Xiaohong Jiang[1*]

[1]State Key Laboratory of Pharmaceutical Biotechnology, Jiangsu Engineering Research Center for MicroRNA Biology and Biotechnology, NJU Advanced Institute for Life Sciences (NAILS), School of life sciences, Nanjing University, 163 Xianlin Road, Nanjing, 210046, China;

[2]Department of Thoracic and Cardiovascular Surgery, the Affiliated Drum Tower Hospital of Nanjing University Medical School, 321 Zhongshan RD, Nanjing, 210008, China.

[3]Genscript Biotech Corporation, Genscript 860 Centennial Ave, Piscataway, NJ 08854, USA.

[*] Correspondences:

Xiaohong Jiang, PhD, Dongjin Wang, PhD, MD or Chen-Yu Zhang, PhD, MD

State Key Laboratory of Pharmaceutical Biotechnology

School of Life Sciences

Nanjing University

Hankou Road 22

Nanjing, Jiangsu 210093

China

E-mail: xiaohongjiang@nju.edu.cn

Supplementary Table 1. Microarray data (Average CT)

Assay	Type	HPT-/AAAD- (CT)	HPT+/AAAD- (CT)	HPT+/AAAD+ (CT)	HPT-/AAAD+ (CT)
ath-miR159a-000338	Target	37	31.8829	37	37
ath-miR159a-4373390	Target	37	37	37	37
dme-miR-7-000268	Target	35.984	36.0073	37	37
hsa-let-7a#-002307	Target	37	37	37	37
hsa-let-7a-4373169	Target	37	37	37	37
hsa-let-7b#-002404	Target	37	37	37	37
hsa-let-7b-4395446	Target	28.9974	27.7593	37	37
hsa-let-7c#-002405	Target	37	37	37	37
hsa-let-7c-4373167	Target	32.951	34.9533	33.9449	37
hsa-let-7d-4395394	Target	29.985	28.9723	31.0316	32.9624
hsa-let-7e#-002407	Target	37	37	37	37
hsa-let-7e-4395517	Target	28.0125	26.863	28.8655	34.074
hsa-let-7f-1#-002417	Target	37	37	37	37
hsa-let-7f-2#-002418	Target	37	37	37	37
hsa-let-7f-4373164	Target	37	37	37	37
hsa-let-7g#-002118	Target	37	37	37	37
hsa-let-7g-4395393	Target	29.9121	30.0207	29.8799	34.0139
hsa-let-7i#-002172	Target	37	37	37	37
hsa-miR-100#-002142	Target	37	37	37	37
hsa-miR-100-4373160	Target	30.9911	31.9412	37	37
hsa-miR-101#-002143	Target	37	37	37	37
hsa-miR-101-4395364	Target	33.8827	33.9386	36.0282	37
hsa-miR-103-4373158	Target	37	37	30.9463	36.9206
hsa-miR-105#-002168	Target	37	37	37	37
hsa-miR-105-4395278	Target	37	37	37	37
hsa-miR-106a#-002170	Target	37	37	37	37
hsa-miR-106a-4395280	Target	26.9581	28.0197	25.9848	31.002
hsa-miR-106b#-002380	Target	37	37	37	37
hsa-miR-106b-4373155	Target	30.9732	31.9763	31.9285	37
hsa-miR-107-4373154	Target	37	37	13.2277	37
hsa-miR-10a#-002288	Target	37	37	37	37
hsa-miR-10a-4373153	Target	32.9446	33.8877	37	37
hsa-miR-10b#-002315	Target	37	36.0014	37	37
hsa-miR-10b-4395329	Target	37	37	37	37
hsa-miR-1178-002777	Target	37	37	37	37
hsa-miR-1179-002776	Target	37	37	37	37
hsa-miR-1180-002847	Target	33.0426	32.9913	37	37
hsa-miR-1182-002830	Target	37	37	37	37
hsa-miR-1183-002841	Target	37	37	37	37
hsa-miR-1184-002842	Target	37	37	37	37
hsa-miR-1197-002810	Target	37	37	37	37

hsa-miR-1200-002829	Target	37	37	37	37
hsa-miR-1201-002781	Target	37	37	37	37
hsa-miR-1203-002877	Target	37	37	37	37
hsa-miR-1204-002872	Target	37	37	37	37
hsa-miR-1205-002778	Target	37	37	37	37
hsa-miR-1206-002878	Target	37	37	37	37
hsa-miR-1208-002880	Target	37	37	37	37
hsa-miR-122#-002130	Target	37	37	37	37
hsa-miR-122-4395356	Target	30.9265	29.0947	31.7878	37
hsa-miR-1224-3P-002752	Target	37	37	37	37
hsa-miR-1225-3P-002766	Target	37	37	37	37
hsa-miR-1226#-002758	Target	37	37	37	37
hsa-miR-1227-002769	Target	35.9413	33.0429	32.0098	37
hsa-miR-1228#-002763	Target	37	37	37	37
hsa-miR-1233-002768	Target	25.8846	25.964	26.9788	37
hsa-miR-1236-002761	Target	37	37	37	37
hsa-miR-1238-002927	Target	37	37	37	37
hsa-miR-124#-002197	Target	37	37	37	37
hsa-miR-1243-002854	Target	11.032	10.9419	37	19.9548
hsa-miR-1244-002791	Target	37	37	37	37
hsa-miR-124-4373295	Target	37	37	37	37
hsa-miR-1245-002823	Target	37	37	37	37
hsa-miR-1247-002893	Target	37	37	30.0424	37
hsa-miR-1248-002870	Target	37	37	37	37
hsa-miR-1249-002868	Target	37	37	37	37
hsa-miR-1250-002887	Target	37	37	37	37
hsa-miR-1251-002820	Target	37	37	37	37
hsa-miR-1252-002860	Target	37	37	37	37
hsa-miR-1253-002894	Target	33.9428	37	37	37
hsa-miR-1254-002818	Target	37	37	33.953	37
hsa-miR-1255A-002805	Target	37	37	37	37
hsa-miR-1255B-002801	Target	32.9848	33.0096	34.0019	37
hsa-miR-1256-002850	Target	37	37	37	37
hsa-miR-1257-002910	Target	37	37	37	37
hsa-miR-1259-002796	Target	37	37	37	37
hsa-miR-125a-3p-4395310	Target	37	35.3903	37	37
hsa-miR-125a-5p-4395309	Target	35.9492	35.0646	35.855	37
hsa-miR-125b-1#-002378	Target	37	36.0182	37	37
hsa-miR-125b-2#-002158	Target	37	37	37	37
hsa-miR-125b-4373148	Target	32.9725	32.9763	34.8568	37
hsa-miR-126#-000451	Target	28.9596	28.9546	27.9517	34.9702
hsa-miR-1260-002896	Target	37	25.9805	37	37
hsa-miR-1262-002852	Target	37	37	37	37
hsa-miR-1263-002784	Target	37	37	37	37

hsa-miR-1264-002799	Target	37	37	37	37
hsa-miR-126-4395339	Target	25.9799	26.102	27.0036	30.9262
hsa-miR-1265-002790	Target	37	34.0012	37	37
hsa-miR-1267-002885	Target	33.9414	37	37	37
hsa-miR-1269-002789	Target	37	37	37	37
hsa-miR-1270-002807	Target	37	37	33.9653	37
hsa-miR-1271-002779	Target	37	37	34.9869	36.1183
hsa-miR-1272-002845	Target	37	37	37	37
hsa-miR-127-3p-4373147	Target	32.9045	34.0095	34.9621	37
hsa-miR-1274A-002883	Target	26.9561	25.9662	27.9951	35.9912
hsa-miR-1274B-002884	Target	20.966	20.9759	22.961	30.9823
hsa-miR-1275-002840	Target	37	37	37	37
hsa-miR-127-5p-4395340	Target	37	6.4436	37	37
hsa-miR-1276-002843	Target	34.9735	34.0271	37	37
hsa-miR-1278-002851	Target	37	37	37	37
hsa-miR-1282-002803	Target	37	37	37	37
hsa-miR-1283-002890	Target	37	37	37	37
hsa-miR-1284-002903	Target	10.1161	37	37	37
hsa-miR-128-4395327	Target	33.0824	31.9838	29.8106	37
hsa-miR-1285-002822	Target	30.0436	28.987	32.044	37
hsa-miR-1286-002773	Target	37	37	37	37
hsa-miR-1288-002832	Target	37	37	37	37
hsa-miR-1289-002871	Target	34.9911	37	37	37
hsa-miR-129#-002298	Target	5.9008	37	37	37
hsa-miR-1290-002863	Target	31.0316	29.9795	25.9396	27.956
hsa-miR-1291-002838	Target	36.0162	37	37	37
hsa-miR-1292-002824	Target	37	37	37	37
hsa-miR-1293-002905	Target	37	37	37	37
hsa-miR-129-3p-4373297	Target	37	37	14.2162	37
hsa-miR-1294-002785	Target	37	37	37	37
hsa-miR-129-5p-4373171	Target	37	6.8804	37	37
hsa-miR-1296-002908	Target	9.7987	37	37	37
hsa-miR-1298-002861	Target	37	37	37	37
hsa-miR-1300-002902	Target	26.4152	33.9503	37	37
hsa-miR-1301-002827	Target	37	37	37	37
hsa-miR-1302-002901	Target	37	37	37	37
hsa-miR-1303-002792	Target	31.8316	29.3086	33.1526	37
hsa-miR-1304-002874	Target	37	37	37	37
hsa-miR-1305-002867	Target	35.9065	37	37	37
hsa-miR-130a#-002131	Target	37	37	37	37
hsa-miR-130a-4373145	Target	30.9614	30.9708	33.9964	37
hsa-miR-130b#-002114	Target	37	37	37	37
hsa-miR-130b-4373144	Target	30.9795	30.9773	33.0234	36.0035
hsa-miR-132#-002132	Target	37	37	37	37

hsa-miR-1324-002815	Target	37	37	37	37
hsa-miR-132-4373143	Target	35.8913	35.1493	36.0299	37
hsa-miR-133a-4395357	Target	31.9458	30.3037	26.9798	32.9552
hsa-miR-133b-4395358	Target	33.9345	37	31.1238	37
hsa-miR-134-4373299	Target	37	37	28.0317	34.8257
hsa-miR-135a-4373140	Target	37	37	37	37
hsa-miR-135b#-002159	Target	37	37	37	37
hsa-miR-135b-4395372	Target	37	37	35.9744	37
hsa-miR-136#-002100	Target	37	37	37	37
hsa-miR-136-4373173	Target	37	37	35.943	37
hsa-miR-137-4373301	Target	37	37	37	37
hsa-miR-138-2#-002144	Target	37	37	37	37
hsa-miR-138-4395395	Target	37	37	37	37
hsa-miR-139-3p-4395424	Target	37	37	32.2376	37
hsa-miR-139-5p-4395400	Target	29.9987	28.8956	28.9771	37
hsa-miR-140-3p-4395345	Target	35.9948	35.7763	30.9794	36.1377
hsa-miR-140-5p-4373374	Target	31.9686	32.0009	30.9976	34.958
hsa-miR-141#-002145	Target	37	37	37	37
hsa-miR-141-4373137	Target	37	37	37	37
hsa-miR-142-3p-4373136	Target	28.9341	28.9295	31.9919	37
hsa-miR-142-5p-4395359	Target	37	36.1583	34.852	37
hsa-miR-143#-002146	Target	37	37	37	37
hsa-miR-143-4395360	Target	35.7889	33.0468	33.8678	37
hsa-miR-1-4395333	Target	35.9267	37	34.993	37
hsa-miR-144#-002148	Target	32.9729	33.9817	33.9749	37
hsa-miR-144-002676	Target	37	37	37	37
hsa-miR-145#-002149	Target	37	37	37	37
hsa-miR-145-4395389	Target	32.0493	30.9997	30.7585	32.8699
hsa-miR-146a#-002163	Target	37	37	37	37
hsa-miR-146a-4373132	Target	28.0492	28.0453	22.9163	27.8492
hsa-miR-146b-3p-4395472	Target	37	37	37	37
hsa-miR-146b-5p-4373178	Target	31.019	30.9247	28.18	33.9902
hsa-miR-147-4373131	Target	37	37	35.9867	37
hsa-miR-147b-4395373	Target	37	37	37	37
hsa-miR-148a#-002134	Target	37	37	37	37
hsa-miR-148a-4373130	Target	32.9301	31.9738	34.0328	37
hsa-miR-148b#-002160	Target	37	36.0471	35.9997	37
hsa-miR-148b-4373129	Target	34.0623	34.9772	34.0342	37
hsa-miR-149#-002164	Target	37	37	37	37
hsa-miR-149-4395366	Target	37	37	37	37
hsa-miR-150-4373127	Target	25.9353	25.945	25.8616	26.9376
hsa-miR-151-3p-002254	Target	29.9583	29.9316	27.9252	32.9178
hsa-miR-151-5P-002642	Target	32.9155	31.9892	32.9987	37
hsa-miR-152-4395170	Target	32.9419	32.9572	31.9622	37

hsa-miR-153-4373305	Target	37	37	37	37
hsa-miR-154#-000478	Target	37	37	37	37
hsa-miR-154-4373270	Target	37	37	37	37
hsa-miR-155#-002287	Target	37	37	37	37
hsa-miR-155-4395459	Target	36.0008	33.862	31.9815	33.9497
hsa-miR-15a#-002419	Target	37	37	35.9485	37
hsa-miR-15a-4373123	Target	33.1421	37	37	37
hsa-miR-15b#-002173	Target	37	37	37	37
hsa-miR-15b-4373122	Target	32.0338	32.0082	33.9832	37
hsa-miR-16-1#-002420	Target	37	37	37	37
hsa-miR-16-2#-002171	Target	37	37	37	37
hsa-miR-16-4373121	Target	25.0045	24.9722	23.9712	28.9431
hsa-miR-17#-002421	Target	37	37	37	37
hsa-miR-17-4395419	Target	27.0005	27.0106	24.9836	30.9164
hsa-miR-181a-2#-002317	Target	37	34.0268	27.038	37
hsa-miR-181a-4373117	Target	32.0502	30.212	31.9765	34.9765
hsa-miR-181c#-002333	Target	37	37	37	37
hsa-miR-181c-4373115	Target	35.3018	37	35.9888	37
hsa-miR-182#-000483	Target	37	37	37	37
hsa-miR-182-4395445	Target	36.0026	37	34.9483	37
hsa-miR-1825-002907	Target	37	37	30.7017	37
hsa-miR-1826-002873	Target	37	37	37	37
hsa-miR-183#-002270	Target	37	37	33.9536	37
hsa-miR-183-4395380	Target	37	37	37	37
hsa-miR-184-4373113	Target	34.9833	32.6779	37	37
hsa-miR-185#-002104	Target	37	37	37	37
hsa-miR-185-4395382	Target	37	31.0481	31.9524	35.9271
hsa-miR-186#-002105	Target	37	37	37	37
hsa-miR-186-4395396	Target	35.0084	32.0475	28.974	32.0244
hsa-miR-187-4373307	Target	37	37	37	37
hsa-miR-188-3p-4395217	Target	37	37	37	37
hsa-miR-18a#-002423	Target	35.9801	36.021	34.95	37
hsa-miR-18a-4395533	Target	30.6415	34.0242	34.1512	37
hsa-miR-18b#-002310	Target	37	37	37	37
hsa-miR-18b-4395328	Target	37	37	37	37
hsa-miR-190-4373110	Target	37	36.0321	37	37
hsa-miR-190b-002263	Target	35.9508	37	34.9438	37
hsa-miR-191#-002678	Target	34.9232	33.9643	34.9697	37
hsa-miR-191-4395410	Target	27.9175	27.922	23.8417	25.9422
hsa-miR-192#-002272	Target	37	37	37	37
hsa-miR-192-4373108	Target	37	35.9714	33.9032	37
hsa-miR-193a-3p-4395361	Target	37	37	37	37
hsa-miR-193a-5p-4395392	Target	32.1102	30.0188	31.8363	36.1575
hsa-miR-193b#-002366	Target	37	37	37	35.9858

hsa-miR-193b-4395478	Target	33.8268	29.8294	30.1748	37
hsa-miR-194#-002379	Target	37	37	37	37
hsa-miR-194-4373106	Target	32.8669	37	36.0384	37
hsa-miR-195#-002107	Target	37	37	37	37
hsa-miR-195-4373105	Target	29.9747	29.9745	29.9615	35.9917
hsa-miR-196a#-002336	Target	37	37	37	37
hsa-miR-196b-4395326	Target	37	37	33.9941	37
hsa-miR-197-4373102	Target	27.0436	26.0585	26.0431	32.0145
hsa-miR-198-4395384	Target	37	37	37	37
hsa-miR-199a-3p-4395415	Target	30.9402	29.9561	29.9004	36.0013
hsa-miR-199a-5p-4373272	Target	37	37	37	37
hsa-miR-199b-5p-4373100	Target	37	37	37	37
hsa-miR-19a#-002424	Target	37	37	37	37
hsa-miR-19a-4373099	Target	30.9077	30.8352	29.0477	33.8151
hsa-miR-19b-1#-002425	Target	36.1522	35.9562	37	37
hsa-miR-19b-4373098	Target	26.062	26.0081	26.0529	32.0226
hsa-miR-200a#-001011	Target	37	37	37	37
hsa-miR-200a-4378069	Target	37	37	33.0867	34.0204
hsa-miR-200b#-002274	Target	37	37	37	37
hsa-miR-200b-4395362	Target	37	37	32.8995	37
hsa-miR-200c#-002286	Target	37	37	37	37
hsa-miR-200c-4395411	Target	33.9116	37	35.2089	37
hsa-miR-202#-002362	Target	37	37	37	37
hsa-miR-202-4395474	Target	37	37	37	37
hsa-miR-203-4373095	Target	36.2283	33.9986	35.9688	37
hsa-miR-204-4373094	Target	32.9523	32.9705	37	37
hsa-miR-205-4373093	Target	37	35.9227	37	37
hsa-miR-206-000510	Target	37	37	28.9079	37
hsa-miR-208-4373091	Target	37	37	37	37
hsa-miR-208b-4395401	Target	37	37	37	37
hsa-miR-20a#-002437	Target	37	37	37	37
hsa-miR-20a-4373286	Target	26.8495	27.0063	26.9229	32.9252
hsa-miR-20b#-002311	Target	37	37	8.8236	37
hsa-miR-20b-4373263	Target	31.0099	30.9549	29.9194	35.0187
hsa-miR-21#-002438	Target	37	37	37	37
hsa-miR-210-4373089	Target	37	37	37	37
hsa-miR-211-4373088	Target	34.8758	37	37	37
hsa-miR-212-4373087	Target	34.685	37	36.0107	34.0417
hsa-miR-213-000516	Target	37	37	37	37
hsa-miR-214#-002293	Target	37	37	37	37
hsa-miR-21-4373090	Target	37	29.8919	37	34.1903
hsa-miR-214-4395417	Target	37	37	36.0942	35.9374
hsa-miR-215-4373084	Target	37	37	37	37
hsa-miR-216a-4395331	Target	37	37	37	37

hsa-miR-216b-4395437	Target	37	37	34.988	37
hsa-miR-217-4395448	Target	37	37	37	37
hsa-miR-218-1#-002094	Target	37	37	37	37
hsa-miR-218-2#-002294	Target	37	37	37	37
hsa-miR-218-4373081	Target	33.9902	33.962	37	37
hsa-miR-219-1-3p-4395206	Target	37	37	37	37
hsa-miR-219-2-3p-4395501	Target	37	37	37	37
hsa-miR-219-5p-4373080	Target	37	37	37	37
hsa-miR-22#-002301	Target	37	37	37	37
hsa-miR-220-4373078	Target	37	37	37	37
hsa-miR-220b-4395317	Target	37	37	37	37
hsa-miR-220c-4395322	Target	37	37	37	37
hsa-miR-221#-002096	Target	37	37	37	37
hsa-miR-221-4373077	Target	28.6779	28.4499	29.0373	32.6635
hsa-miR-222#-002097	Target	37	37	37	37
hsa-miR-222-4395387	Target	29.9906	29.9798	25.9567	30.9625
hsa-miR-223#-002098	Target	31.04	30.9816	29.9936	32.9771
hsa-miR-223-4395406	Target	22.9545	22.9374	21.9725	26.9519
hsa-miR-22-4373079	Target	37	10.1116	37	37
hsa-miR-224-4395210	Target	33.9833	32.9925	32.9569	37
hsa-miR-23a#-002439	Target	35.9513	35.9712	37	37
hsa-miR-23a-4373074	Target	37	37	37	37
hsa-miR-23b#-002126	Target	37	37	37	37
hsa-miR-23b-4373073	Target	37	37	37	37
hsa-miR-24-1#-002440	Target	37	37	37	37
hsa-miR-24-2#-002441	Target	37	37	37	37
hsa-miR-24-4373072	Target	25.9869	27.0127	23.9282	27.9862
hsa-miR-25#-002442	Target	37	37	37	37
hsa-miR-25-4373071	Target	30.94	32.0245	29.9624	29.5728
hsa-miR-26a-1#-002443	Target	36.0311	32.9959	32.9663	36.0129
hsa-miR-26a-2#-002115	Target	37	37	37	37
hsa-miR-26a-4395166	Target	29.9376	30.0368	29.9611	34.9508
hsa-miR-26b#-002444	Target	37	36.9578	34.9336	37
hsa-miR-26b-4395167	Target	31.0133	30.9579	31.9774	34.9119
hsa-miR-27a#-002445	Target	35.9456	33.9808	36.0126	37
hsa-miR-27a-4373287	Target	31.9538	32.0041	32.9663	33.9289
hsa-miR-27b#-002174	Target	37	37	37	37
hsa-miR-27b-4373068	Target	32.0463	31.8868	31.9272	32.9056
hsa-miR-28-3p-4395557	Target	29.9566	30.0061	28.8906	34.0296
hsa-miR-28-5p-4373067	Target	32.9727	32.9547	34.0352	34.8139
hsa-miR-296-3p-4395212	Target	37	37	37	37
hsa-miR-296-5p-4373066	Target	31.9918	33.0198	32.943	37
hsa-miR-298-4395301	Target	37	37	37	37
hsa-miR-299-3p-4373189	Target	37	37	37	37

hsa-miR-299-5p-4373188	Target	37	37	37	37
hsa-miR-29a#-002447	Target	37	37	37	37
hsa-miR-29a-4395223	Target	32.941	32.9778	31.9415	37
hsa-miR-29b-1#-002165	Target	37	37	37	37
hsa-miR-29b-2#-002166	Target	37	37	37	37
hsa-miR-29b-4373288	Target	37	37	34.9752	34.9706
hsa-miR-29c-4395171	Target	37	37	35.9415	37
hsa-miR-301a-4373064	Target	36.0217	32.9384	33.0121	37
hsa-miR-301b-4395503	Target	37	35.9328	37	37
hsa-miR-302a#-002381	Target	37	37	37	37
hsa-miR-302a-4378070	Target	37	37	37	37
hsa-miR-302b#-002119	Target	37	37	37	37
hsa-miR-302b-4378071	Target	37	37	37	37
hsa-miR-302c#-000534	Target	37	37	37	37
hsa-miR-302c-4378072	Target	16.13	15.3673	22.001	30.0056
hsa-miR-302d#-002120	Target	37	37	37	37
hsa-miR-302d-000535	Target	37	37	36.0114	37
hsa-miR-30a-3p-000416	Target	30.958	33.0585	30.9598	37
hsa-miR-30a-5p-000417	Target	27.9539	27.9638	27.9423	33.9338
hsa-miR-30b#-002129	Target	37	37	37	37
hsa-miR-30b-4373290	Target	27.9889	28.0314	29.9852	37
hsa-miR-30c-1#-002108	Target	37	37	37	37
hsa-miR-30c-2#-002110	Target	37	37	37	37
hsa-miR-30c-4373060	Target	27.9537	27.9271	28.9388	32.9942
hsa-miR-30d#-002305	Target	37	37	37	37
hsa-miR-30d-000420	Target	30.0031	30.9495	30.9858	37
hsa-miR-30e-3p-000422	Target	31.9459	30.944	30.967	37
hsa-miR-31#-002113	Target	37	37	37	37
hsa-miR-31-4395390	Target	37	37	33.261	37
hsa-miR-32#-002111	Target	37	37	37	37
hsa-miR-320-4395388	Target	26.0394	25.9535	23.9986	27.9523
hsa-miR-320B-002844	Target	32.9803	30.9746	30.9953	37
hsa-miR-323-3p-4395338	Target	37	37	35.9827	37
hsa-miR-32-4395220	Target	37	37	37	37
hsa-miR-324-3p-4395272	Target	32.9746	34.014	32.1566	37
hsa-miR-324-5p-4373052	Target	33.9889	32.98	33.9871	37
hsa-miR-325-4373051	Target	37	37	37	37
hsa-miR-326-4373050	Target	37	37	37	37
hsa-miR-328-4373049	Target	27.8514	28.9928	29.9925	37
hsa-miR-329-4373191	Target	37	37	37	37
hsa-miR-330-3p-4373047	Target	37	37	37	37
hsa-miR-330-5p-4395341	Target	37	37	37	37
hsa-miR-331-3p-4373046	Target	29.1162	30.2404	30.1521	37
hsa-miR-331-5p-4395344	Target	37	37	37	37

hsa-miR-335#-002185	Target	37	37	37	37
hsa-miR-335-4373045	Target	32.9892	31.9429	31.9392	37
hsa-miR-337-3p-002157	Target	37	37	37	37
hsa-miR-337-5p-4395267	Target	37	37	37	37
hsa-miR-338-3p-4395363	Target	37	37	37	37
hsa-miR-338-5P-002658	Target	30.9858	31.9514	35.0531	37
hsa-miR-339-3p-4395295	Target	33.0724	34.7444	29.9715	33.9731
hsa-miR-339-5p-4395368	Target	37	37	37	37
hsa-miR-33a#-002136	Target	37	37	37	37
hsa-miR-33a-002135	Target	37	37	37	37
hsa-miR-33b-4395196	Target	37	37	37	37
hsa-miR-340#-002259	Target	35.9913	33.9456	32.925	37
hsa-miR-340-4395369	Target	37	36.0201	35.9766	37
hsa-miR-342-3p-4395371	Target	29.0143	28.9263	25.9122	29.0007
hsa-miR-342-5p-4395258	Target	37	37	37	37
hsa-miR-345-4395297	Target	32.9474	33.0103	31.9586	35.992
hsa-miR-346-4373038	Target	37	37	37	37
hsa-miR-34a#-002316	Target	37	37	35.6686	37
hsa-miR-34a-4395168	Target	37	37	36.1056	37
hsa-miR-34b-000427	Target	37	37	37	37
hsa-miR-34b-002102	Target	31.9788	30.9456	32.1568	37
hsa-miR-34c-5p-4373036	Target	37	37	37	37
hsa-miR-361-3p-002116	Target	37	37	37	37
hsa-miR-361-5p-4373035	Target	36.0572	37	37	37
hsa-miR-362-3p-4395228	Target	37	37	37	37
hsa-miR-362-5p-4378092	Target	34.9846	37	37	37
hsa-miR-363#-001283	Target	37	37	37	37
hsa-miR-363-4378090	Target	37	37	37	37
hsa-miR-365-4373194	Target	37	37	37	37
hsa-miR-367#-002121	Target	37	37	37	37
hsa-miR-367-4373034	Target	31.0755	37	30.7976	37
hsa-miR-369-3p-4373032	Target	37	37	37	37
hsa-miR-369-5p-4373195	Target	37	37	37	37
hsa-miR-370-4395386	Target	37	37	37	37
hsa-miR-371-3p-4395235	Target	37	37	37	37
hsa-miR-372-4373029	Target	37	37	37	37
hsa-miR-373-4378073	Target	37	37	37	37
hsa-miR-374a#-002125	Target	37	37	37	37
hsa-miR-374a-4373028	Target	33.9793	31.8596	32.8571	35.9631
hsa-miR-374b#-002391	Target	37	37	37	37
hsa-miR-374b-4381045	Target	30.9803	30.9278	30.9851	37
hsa-miR-375-4373027	Target	29.9655	30.9709	32.9642	37
hsa-miR-376a#-002127	Target	37	37	37	37
hsa-miR-376a-4373026	Target	34.0933	33.9757	32.9941	33.9886

hsa-miR-376b-4373196	Target	28.1339	37	27.9889	31.8258
hsa-miR-376c-4395233	Target	34.0084	32.9732	31.9697	37
hsa-miR-377#-002128	Target	37	37	37	37
hsa-miR-377-4373025	Target	23.9813	23.9286	23.9966	28.9449
hsa-miR-378-000567	Target	32.9966	34.9603	34.0338	37
hsa-miR-378-002243	Target	37	37	37	37
hsa-miR-379-4373349	Target	37	34.0699	35.9566	37
hsa-miR-380-4373022	Target	37	37	37	37
hsa-miR-380-5p-000570	Target	36.305	37	37	37
hsa-miR-381-4373020	Target	20.0653	19.9559	25.1119	28.9653
hsa-miR-382-4373019	Target	37	37	37	37
hsa-miR-383-4373018	Target	37	37	37	37
hsa-miR-384-4373017	Target	37	37	37	37
hsa-miR-409-3p-002332	Target	31.9923	32.0119	27.9926	31.9933
hsa-miR-409-5p-4395442	Target	37	37	37	37
hsa-miR-410-4378093	Target	37	36.178	36.0016	37
hsa-miR-411#-002238	Target	37	37	37	37
hsa-miR-411-4381013	Target	37	37	24.2943	35.9675
hsa-miR-412-4373199	Target	37	37	37	37
hsa-miR-422a-4395408	Target	33.6693	37	37	37
hsa-miR-423-5p-4395451	Target	31.1477	37	30.1866	34.9769
hsa-miR-424#-002309	Target	37	37	37	37
hsa-miR-424-4373201	Target	37	37	37	37
hsa-miR-425#-002302	Target	37	34.9763	37	37
hsa-miR-425-4380926	Target	28.1157	37	28.764	32.9423
hsa-miR-429-4373203	Target	37	37	37	37
hsa-miR-431#-002312	Target	37	37	37	37
hsa-miR-431-4395173	Target	37	37	37	37
hsa-miR-432#-001027	Target	37	37	37	37
hsa-miR-432-001026	Target	31.8933	33.9437	27.9671	30.916
hsa-miR-433-4373205	Target	31.0547	31.0364	32.9642	37
hsa-miR-448-4373206	Target	37	37	37	37
hsa-miR-449a-4373207	Target	37	37	37	37
hsa-miR-449b-4381011	Target	35.9772	37	37	37
hsa-miR-450a-4395414	Target	37	37	37	37
hsa-miR-450b-3p-4395319	Target	37	37	37	37
hsa-miR-450b-5p-4395318	Target	37	37	33.9748	37
hsa-miR-451-4373360	Target	26.9372	27.9385	28.9645	35.9867
hsa-miR-452#-002330	Target	37	37	31.1801	37
hsa-miR-452-4395440	Target	37	37	34.9661	37
hsa-miR-453-4395429	Target	37	37	37	37
hsa-miR-454#-001996	Target	37	37	32.9866	37
hsa-miR-454-4395434	Target	32.973	32.9812	28.9266	35.9454
hsa-miR-455-3p-4395355	Target	37	37	37	37

hsa-miR-455-5p-4378098	Target	37	37	37	37
hsa-miR-483-3p-002339	Target	37	37	37	37
hsa-miR-483-5p-4395449	Target	30.0034	29.9691	27.9433	29.9583
hsa-miR-484-4381032	Target	24.9629	23.9506	20.9427	26.9301
hsa-miR-485-3p-4378095	Target	30.9607	31.1662	32.9292	37
hsa-miR-485-5p-4373212	Target	37	37	37	37
hsa-miR-486-3p-4395204	Target	34.7868	37	31.9526	37
hsa-miR-486-5p-4378096	Target	26.0836	29.0621	25.9755	29.9922
hsa-miR-487a-4378097	Target	37	37	37	37
hsa-miR-487b-4378102	Target	37	35.8884	37	37
hsa-miR-488-001106	Target	37	37	35.9956	37
hsa-miR-488-4395468	Target	37	37	37	37
hsa-miR-489-4395469	Target	37	37	37	37
hsa-miR-490-3p-4373215	Target	37	37	37	37
hsa-miR-491-3p-4395471	Target	37	37	37	37
hsa-miR-491-5p-4381053	Target	33.9777	37	34.9599	37
hsa-miR-492-4373217	Target	37	37	37	37
hsa-miR-493-4395475	Target	37	37	37	37
hsa-miR-494-4395476	Target	37	36.1398	35.9822	37
hsa-miR-495-4381078	Target	32.9491	32.9228	32.9974	37
hsa-miR-496-4386771	Target	37	37	37	37
hsa-miR-497#-002368	Target	37	37	37	37
hsa-miR-497-001043	Target	37	37	37	37
hsa-miR-499-3p-4395538	Target	37	37	37	37
hsa-miR-499-5p-4381047	Target	37	37	37	37
hsa-miR-500-001046	Target	37	37	37	37
hsa-miR-500-4395539	Target	33.9917	37	37	37
hsa-miR-501-3p-4395546	Target	37	37	37	37
hsa-miR-501-5p-4373226	Target	37	37	33.0244	37
hsa-miR-502-3p-4395194	Target	37	37	37	37
hsa-miR-502-5p-4373227	Target	37	35.9501	37	37
hsa-miR-503-4373228	Target	37	37	37	37
hsa-miR-504-4395195	Target	37	37	37	37
hsa-miR-505#-002087	Target	32.9427	32.9774	31.9687	37
hsa-miR-505-4395200	Target	37	37	37	37
hsa-miR-506-4373231	Target	37	37	37	37
hsa-miR-507-4373232	Target	37	37	37	37
hsa-miR-508-3p-4373233	Target	37	37	37	37
hsa-miR-508-5p-4395203	Target	37	37	37	37
hsa-miR-509-3-5p-4395266	Target	37	37	37	37
hsa-miR-509-5p-4395346	Target	37	37	37	37
hsa-miR-510-4395352	Target	37	37	37	37
hsa-miR-511-4373236	Target	37	36.0867	35.943	37
hsa-miR-512-3p-4381034	Target	37	37	37	37

hsa-miR-512-5p-4373238	Target	37	37	37	37
hsa-miR-513-5p-4395201	Target	37	37	37	37
hsa-miR-513B-002757	Target	37	37	37	37
hsa-miR-513C-002756	Target	37	37	37	37
hsa-miR-515-3p-4395480	Target	21.0417	20.1739	24.0687	24.8789
hsa-miR-515-5p-4373242	Target	37	37	37	37
hsa-miR-516-3p-001149	Target	37	36.9609	36.9774	37
hsa-miR-516a-5p-4395527	Target	37	34.9309	37	37
hsa-miR-516b-4395172	Target	37	37	37	37
hsa-miR-517#-001113	Target	37	37	37	37
hsa-miR-517a-4395513	Target	37	37	36.038	34.9215
hsa-miR-517b-4373244	Target	37	37	37	37
hsa-miR-517c-4373264	Target	37	37	37	37
hsa-miR-518a-3p-4395508	Target	37	37	37	37
hsa-miR-518a-5p-4395507	Target	37	37	37	37
hsa-miR-518b-4373246	Target	37	37	35.9744	37
hsa-miR-518c#-001158	Target	37	37	37	37
hsa-miR-518c-4395512	Target	37	37	37	37
hsa-miR-518d-3p-4373248	Target	37	37	37	37
hsa-miR-518d-5p-4395500	Target	37	37	37	37
hsa-miR-518e#-002371	Target	37	37	37	37
hsa-miR-518e-4395506	Target	37	37	37	37
hsa-miR-518f#-002387	Target	37	37	37	37
hsa-miR-518f-4395499	Target	35.9666	30.9543	30.9135	32.9495
hsa-miR-519a-4395526	Target	37	37	37	37
hsa-miR-519b-3p-002384	Target	37	37	37	37
hsa-miR-519c-3p-4373251	Target	37	37	37	37
hsa-miR-519d-4395514	Target	37	37	37	37
hsa-miR-519e#-001166	Target	37	37	37	37
hsa-miR-519e-4395481	Target	37	37	37	37
hsa-miR-520a-3p-4373268	Target	37	37	37	37
hsa-miR-520a-5p-4378085	Target	37	37	37	37
hsa-miR-520b-4373252	Target	37	37	36.1529	37
hsa-miR-520c-3p-002400	Target	37	36.272	37	34.9878
hsa-miR-520D-3P-002743	Target	37	34.9705	37	37
hsa-miR-520d-5p-4395504	Target	37	37	37	37
hsa-miR-520e-4373255	Target	37	28.3461	37	37
hsa-miR-520f-4373256	Target	35.017	37	37	37
hsa-miR-520g-4373257	Target	37	37	37	37
hsa-miR-520h-001170	Target	37	37	37	37
hsa-miR-521-4373259	Target	36.1047	37	37	37
hsa-miR-522-4395524	Target	37	37	37	37
hsa-miR-523-4395497	Target	37	37	37	37
hsa-miR-524-001173	Target	37	37	37	37

hsa-miR-524-5p-4395174	Target	37	37	37	37
hsa-miR-525-3p-4395496	Target	37	37	37	37
hsa-miR-525-5p-4378088	Target	37	37	37	37
hsa-miR-526b-4395493	Target	37	37	37	37
hsa-miR-532-3p-4395466	Target	36.1465	33.8253	34.077	37
hsa-miR-532-5p-4380928	Target	36.0734	35.8808	35.9692	37
hsa-miR-539-4378103	Target	34.9792	33.9696	34.9774	37
hsa-miR-541#-002200	Target	37	37	37	37
hsa-miR-541-4395312	Target	37	37	37	37
hsa-miR-542-3p-4378101	Target	37	37	37	37
hsa-miR-542-5p-4395351	Target	37	37	37	37
hsa-miR-543-002376	Target	33.1833	34.0302	37	37
hsa-miR-544-4395376	Target	37	37	37	37
hsa-miR-545#-002266	Target	37	37	37	37
hsa-miR-545-4395378	Target	37	33.932	37	37
hsa-miR-548a-3p-4380948	Target	37	37	35.942	37
hsa-miR-548a-5p-4395523	Target	37	37	37	37
hsa-miR-548b-3p-4380951	Target	37	37	37	37
hsa-miR-548b-5p-4395519	Target	37	34.0312	37	37
hsa-miR-548c-3p-4380993	Target	37	37	37	37
hsa-miR-548c-5p-4395540	Target	37	34.9109	37	37
hsa-miR-548d-3p-4381008	Target	37	37	37	37
hsa-miR-548d-5p-4395348	Target	34.0686	33.9564	37	37
hsa-miR-548E-002881	Target	37	37	37	37
hsa-miR-548G-002879	Target	37	37	37	37
hsa-miR-548H-002816	Target	37	37	37	37
hsa-miR-548I-002909	Target	37	37	37	37
hsa-miR-548J-002783	Target	37	37	37	37
hsa-miR-548K-002819	Target	37	37	37	37
hsa-miR-548L-002904	Target	37	37	37	37
hsa-miR-548M-002775	Target	37	37	37	37
hsa-miR-548N-002888	Target	37	37	37	37
hsa-miR-548P-002798	Target	37	37	37	37
hsa-miR-549-001511	Target	37	37	37	37
hsa-miR-550-001544	Target	24.7206	37	37	37
hsa-miR-550-002410	Target	37	37	37	37
hsa-miR-551a-001519	Target	37	37	37	37
hsa-miR-551b#-002346	Target	37	37	37	37
hsa-miR-551b-4380945	Target	37	37	37	37
hsa-miR-552-001520	Target	37	37	37	37
hsa-miR-553-001521	Target	37	37	34.9514	37
hsa-miR-554-001522	Target	37	37	37	37
hsa-miR-555-001523	Target	37	37	37	37
hsa-miR-556-3p-4395456	Target	37	37	37	37

hsa-miR-556-5p-4395455	Target	37	37	37	37
hsa-miR-557-001525	Target	37	37	37	37
hsa-miR-558-001526	Target	37	37	37	37
hsa-miR-559-001527	Target	37	37	37	37
hsa-miR-561-4380938	Target	37	37	37	37
hsa-miR-562-001529	Target	37	37	37	37
hsa-miR-563-001530	Target	37	37	37	37
hsa-miR-564-001531	Target	37	37	37	37
hsa-miR-566-001533	Target	37	37	27.8496	37
hsa-miR-567-001534	Target	37	37	37	37
hsa-miR-569-001536	Target	37	37	37	37
hsa-miR-570-4395458	Target	37	37	37	37
hsa-miR-571-001613	Target	28.8234	37	29.0478	36.0776
hsa-miR-572-001614	Target	34.9513	36.1936	37	37
hsa-miR-573-001615	Target	37	37	37	37
hsa-miR-574-3p-4395460	Target	28.9799	28.9908	25.9723	28.9549
hsa-miR-575-001617	Target	37	37	37	37
hsa-miR-576-3p-4395462	Target	37	37	37	37
hsa-miR-576-5p-4395461	Target	37	37	37	37
hsa-miR-577-002675	Target	37	37	37	37
hsa-miR-578-001619	Target	37	37	37	37
hsa-miR-579-4395509	Target	37	37	37	37
hsa-miR-580-001621	Target	34.8124	37	37	37
hsa-miR-581-001622	Target	37	37	37	37
hsa-miR-582-3p-4395510	Target	37	37	37	37
hsa-miR-582-5p-4395175	Target	37	37	37	37
hsa-miR-583-001623	Target	37	37	37	37
hsa-miR-584-001624	Target	37	37	37	33.9787
hsa-miR-585-001625	Target	37	37	37	37
hsa-miR-586-001539	Target	37	37	37	37
hsa-miR-587-001540	Target	37	37	37	37
hsa-miR-588-001542	Target	37	37	37	37
hsa-miR-589-001543	Target	37	37	31.9518	37
hsa-miR-589-4395520	Target	35.8743	37	37	37
hsa-miR-590-3P-002677	Target	36.946	36.9613	35.9795	37
hsa-miR-590-5p-4395176	Target	32.9457	35.947	31.9536	37
hsa-miR-591-001545	Target	33.9853	37	34.89	37
hsa-miR-592-001546	Target	37	37	37	37
hsa-miR-593-001547	Target	37	37	37	37
hsa-miR-593-002411	Target	37	37	37	37
hsa-miR-595-001987	Target	37	37	37	37
hsa-miR-596-001550	Target	37	37	37	37
hsa-miR-597-4380960	Target	33.9803	33.9601	37	37
hsa-miR-598-4395179	Target	37	34.9921	37	37

hsa-miR-599-001554	Target	37	37	37	37
hsa-miR-600-001556	Target	37	37	37	37
hsa-miR-601-001558	Target	37	35.0061	37	37
hsa-miR-603-001566	Target	37	37	37	34.9367
hsa-miR-604-001567	Target	37	37	5.3173	37
hsa-miR-605-001568	Target	37	32.7416	37	37
hsa-miR-606-001569	Target	37	37	37	37
hsa-miR-607-001570	Target	37	37	37	37
hsa-miR-608-001571	Target	37	37	37	37
hsa-miR-609-001573	Target	37	37	37	37
hsa-miR-613-001586	Target	37	37	37	37
hsa-miR-614-001587	Target	36.724	37	37	37
hsa-miR-615-3p-4386777	Target	37	37	37	37
hsa-miR-615-5p-4395464	Target	37	37	37	37
hsa-miR-616-001589	Target	37	37	37	37
hsa-miR-616-4395525	Target	37	37	37	37
hsa-miR-617-001591	Target	37	37	37	37
hsa-miR-618-4380996	Target	37	37	37	37
hsa-miR-620-002672	Target	37	37	37	37
hsa-miR-621-001598	Target	37	37	37	37
hsa-miR-622-001553	Target	37	37	37	37
hsa-miR-623-001555	Target	37	37	37	32.1133
hsa-miR-624-001557	Target	37	37	37	37
hsa-miR-624-4395541	Target	37	37	37	37
hsa-miR-625#-002432	Target	30.9854	31.9789	25.9356	32.9272
hsa-miR-625-4395542	Target	37	37	37	37
hsa-miR-626-001559	Target	37	37	37	37
hsa-miR-627-4380967	Target	37	37	37	37
hsa-miR-628-3p-002434	Target	37	37	32.9995	34.9865
hsa-miR-628-5p-4395544	Target	33.9509	37	34.978	37
hsa-miR-629-001562	Target	35.0242	33.9561	33.9766	32.9873
hsa-miR-629-4395547	Target	34.9409	33.9515	34.9958	37
hsa-miR-630-001563	Target	37	37	37	37
hsa-miR-631-001564	Target	37	37	37	37
hsa-miR-633-001574	Target	37	37	37	37
hsa-miR-634-001576	Target	37	37	30.9143	37
hsa-miR-635-001578	Target	35.3206	37	37	37
hsa-miR-636-4395199	Target	31.9253	32.0833	32.8929	37
hsa-miR-637-001581	Target	30.834	37	37	37
hsa-miR-638-001582	Target	31.0405	30.0422	37	37
hsa-miR-639-001583	Target	36.1898	37	37	37
hsa-miR-640-001584	Target	37	37	37	37
hsa-miR-641-001585	Target	31.3387	37	37	37
hsa-miR-642-4380995	Target	32.9626	34.965	37	37

hsa-miR-643-001594	Target	37	37	37	37
hsa-miR-644-001596	Target	37	37	37	37
hsa-miR-645-001597	Target	35.9746	33.9666	37	37
hsa-miR-646-001599	Target	37	37	37	37
hsa-miR-647-001600	Target	37	37	37	37
hsa-miR-648-001601	Target	37	37	37	37
hsa-miR-649-001602	Target	37	37	37	37
hsa-miR-650-001603	Target	37	37	37	37
hsa-miR-651-4381007	Target	37	37	37	37
hsa-miR-652-4395463	Target	33.1422	34.9827	35.0121	37
hsa-miR-653-4395403	Target	37	37	37	37
hsa-miR-654-3p-4395350	Target	37	37	37	37
hsa-miR-654-5p-4381014	Target	37	37	37	37
hsa-miR-655-4381015	Target	37	37	37	37
hsa-miR-656-001510	Target	37	37	37	37
hsa-miR-657-001512	Target	37	37	37	37
hsa-miR-658-001513	Target	37	37	37	37
hsa-miR-659-001514	Target	37	37	37	37
hsa-miR-660-4380925	Target	29.9388	31.1941	31.9269	37
hsa-miR-661-001606	Target	37	37	37	37
hsa-miR-662-001607	Target	37	37	34.0045	37
hsa-miR-663B-002857	Target	37	37	37	37
hsa-miR-664-002897	Target	37	37	37	37
hsa-miR-665-002681	Target	37	37	37	37
hsa-miR-668-001992	Target	37	37	37	37
hsa-miR-671-3p-4395433	Target	37	33.0206	34.0004	37
hsa-miR-672-4395438	Target	37	34.1983	37	37
hsa-miR-674-4395193	Target	37	37	37	37
hsa-miR-675-002005	Target	37	37	37	37
hsa-miR-708#-002342	Target	37	37	37	37
hsa-miR-708-4395452	Target	37	37	37	37
hsa-miR-7-2#-002314	Target	31.8371	37	10.8135	37
hsa-miR-720-002895	Target	27.0115	26.9901	25.9568	30.9565
hsa-miR-744#-002325	Target	37	33.9088	32.5653	37
hsa-miR-744-4395435	Target	31.9938	30.9679	30.9712	37
hsa-miR-758-4395180	Target	37	37	36.0025	37
hsa-miR-765-002643	Target	37	37	37	37
hsa-miR-766-001986	Target	27.2529	29.0202	26.0093	31.9397
hsa-miR-767-3p-001995	Target	37	37	37	37
hsa-miR-767-5p-001993	Target	37	37	37	37
hsa-miR-769-3p-002003	Target	37	37	37	37
hsa-miR-769-5p-001998	Target	33.0963	37	34.9943	37
hsa-miR-770-5p-002002	Target	37	37	37	37
hsa-miR-802-002004	Target	37	37	37	37

hsa-miR-871-4395465	Target	37	37	37	37
hsa-miR-872-4395375	Target	37	37	37	37
hsa-miR-873-4395467	Target	37	37	37	37
hsa-miR-874-4395379	Target	37	37	30.7746	35.9461
hsa-miR-875-3p-4395315	Target	37	37	37	37
hsa-miR-875-5p-002203	Target	37	37	37	37
hsa-miR-876-3p-4395336	Target	37	37	37	37
hsa-miR-876-5p-4395316	Target	37	37	37	37
hsa-miR-885-3p-4395483	Target	37	37	37	37
hsa-miR-885-5p-4395407	Target	29.9861	27.9423	27.9897	35.9526
hsa-miR-886-3p-4395305	Target	31.9154	32.9861	35.0105	37
hsa-miR-886-5p-4395304	Target	36.0202	36.9476	36.0244	37
hsa-miR-887-4395485	Target	37	37	37	37
hsa-miR-888#-002213	Target	37	37	37	37
hsa-miR-888-4395323	Target	37	37	37	37
hsa-miR-889-4395313	Target	37	37	37	37
hsa-miR-890-4395320	Target	37	37	37	35.033
hsa-miR-891a-4395302	Target	37	37	37	37
hsa-miR-891b-4395321	Target	37	37	37	37
hsa-miR-892a-4395306	Target	37	37	37	37
hsa-miR-892b-002214	Target	37	37	37	32.9311
hsa-miR-9#-002231	Target	37	35.9307	37	37
hsa-miR-920-002150	Target	37	37	37	37
hsa-miR-921-002151	Target	37	37	37	37
hsa-miR-922-002152	Target	37	37	37	37
hsa-miR-924-002154	Target	37	37	37	37
hsa-miR-92a-1#-002137	Target	37	37	37	37
hsa-miR-92a-2#-002138	Target	37	37	37	37
hsa-miR-92a-4395169	Target	24.7463	25.1908	26.0077	32.113
hsa-miR-92b#-002343	Target	37	37	37	37
hsa-miR-93#-002139	Target	35.0142	33.023	29.9322	34.9755
hsa-miR-933-002176	Target	37	37	37	37
hsa-miR-934-002177	Target	37	37	37	37
hsa-miR-93-4373302	Target	28.3052	28.9026	29.0235	35.0316
hsa-miR-935-002178	Target	37	37	37	37
hsa-miR-936-002179	Target	37	37	37	37
hsa-miR-937-002180	Target	37	37	37	37
hsa-miR-938-002181	Target	37	37	37	37
hsa-miR-939-002182	Target	37	37	37	36.1193
hsa-miR-941-002183	Target	37	37	37	37
hsa-miR-942-002187	Target	33.9774	33.7085	33.7684	36.0186
hsa-miR-943-002188	Target	37	37	37	37
hsa-miR-9-4373285	Target	33.9808	37	37	37
hsa-miR-944-002189	Target	37	37	37	37

hsa-miR-95-4373011	Target	37	37	37	37
hsa-miR-96#-002140	Target	37	37	37	37
hsa-miR-96-4373372	Target	37	37	37	37
hsa-miR-98-4373009	Target	37	37	37	37
hsa-miR-99a#-002141	Target	37	37	37	37
hsa-miR-99a-4373008	Target	30.8571	29.5029	27.3349	37
hsa-miR-99b#-002196	Target	37	37	33.0454	37
hsa-miR-99b-4373007	Target	29.9177	32.0484	33.0155	37
MammU6-4395470	Candidate Control	24.7382	22.8592	23.5298	24.3349
mmu-let-7d#-001178	Target	37	37	37	37
rno-miR-29c#-001818	Target	37	37	37	37
rno-miR-7#-001338	Target	37	37	31.0731	35.9831
RNU44-001094	Target	37	37	37	37
RNU44-4373384	Target	37	24	37	37
RNU48-001006	Target	31.9892	31.8997	29.2482	30.9765
RNU48-4373383	Target	33.0253	31.9494	30.0043	31.971
U6 snRNA-001973	Candidate Control	24.2352	22.9613	23.5567	23.4088

Supplementary Table 2. Differentially expressed miRNAs in HPT+/AAAD+ serum samples compared to HPT+/AAAD- serum samples determined by TLDA

MiRNAs	Ct of HPT+/AAAD-	Ct of HPT+/AAAD+	Fold changes
hsa-miR-181a-2*	34.0	27.0	127.010
hsa-miR-625*	32.0	25.9	65.950
hsa-miR-432	33.9	28.0	62.970
hsa-miR-146a	28.0	22.9	34.993
hsa-miR-140-3p	35.8	31.0	27.798
hsa-miR-339-3p	34.7	30.0	27.339
hsa-miR-191	27.9	23.8	16.916
hsa-miR-454	33.0	28.9	16.617
hsa-miR-1290	30.0	25.9	16.449
hsa-miR-222	30.0	26.0	16.258
hsa-miR-409-3p	32.0	28.0	16.215
hsa-miR-590-5p	35.9	32.0	15.927
hsa-miR-133a	30.3	27.0	10.014
hsa-miR-93*	33.0	29.9	8.520
hsa-miR-486-5p	29.1	26.0	8.495
hsa-miR-24	27.0	23.9	8.483
hsa-miR-186	32.0	29.0	8.418
hsa-miR-574-3p	29.0	26.0	8.103
hsa-miR-342-3p	28.9	25.9	8.079
hsa-miR-766	29.0	26.0	8.061
hsa-miR-484	24.0	20.9	8.044
hsa-miR-146b-5p	30.9	28.2	6.703
hsa-miR-128	32.0	29.8	4.510
hsa-miR-99a	29.5	27.3	4.494
hsa-miR-30a-3p	33.1	31.0	4.283
hsa-miR-192	36.0	33.9	4.194
hsa-miR-25	32.0	30.0	4.176
hsa-miR-106a	28.0	26.0	4.098
hsa-miR-17	27.0	25.0	4.076
hsa-miR-483-5p	30.0	27.9	4.072
hsa-miR-26b*	37.0	34.9	4.068
hsa-miR-151-3p	29.9	27.9	4.018
hsa-miR-320	26.0	24.0	3.877
hsa-miR-155	33.9	32.0	3.682
hsa-miR-324-3p	34.0	32.2	3.624
hsa-miR-19a	30.8	29.0	3.452
hsa-miR-744*	33.9	32.6	2.538
hsa-miR-142-5p	36.2	34.9	2.473
hsa-miR-28-3p	30.0	28.9	2.167
hsa-miR-18a*	36.0	35.0	2.101
hsa-miR-345	33.0	32.0	2.073

hsa-miR-29a	33.0	31.9	2.051
hsa-miR-20b	31.0	29.9	2.050
hsa-miR-720	27.0	26.0	2.047
hsa-miR-1227	33.0	32.0	2.046
hsa-miR-340*	33.9	32.9	2.029
hsa-miR-505*	33.0	32.0	2.012
hsa-let-7c	35.0	33.9	2.012
hsa-miR-376c	33.0	32.0	2.005
hsa-miR-140-5p	32.0	31.0	2.005
hsa-miR-126*	29.0	28.0	2.004
hsa-miR-16	25.0	24.0	2.001
hsa-miR-152	33.0	32.0	1.993
hsa-miR-223*	31.0	30.0	1.983
hsa-miR-590-3p	37.0	36.0	1.975
hsa-miR-376a	34.0	33.0	1.975
hsa-miR-223	22.9	22.0	1.952
hsa-miR-148b	35.0	34.0	1.923
hsa-miR-378	35.0	34.0	1.901
hsa-miR-886-5p	36.9	36.0	1.896
hsa-miR-145	31.0	30.8	1.182
hsa-miR-410	36.2	36.0	1.130
hsa-miR-494	36.1	36.0	1.115
hsa-miR-511	36.1	35.9	1.105
hsa-let-7g	30.0	29.9	1.103
hsa-miR-331-3p	30.2	30.2	1.063
hsa-miR-20a	27.0	26.9	1.060
hsa-miR-150	25.9	25.9	1.060
hsa-miR-296-5p	33.0	32.9	1.055
hsa-miR-26a	30.0	30.0	1.054
hsa-miR-199a-3p	30.0	29.9	1.039
hsa-miR-106b	32.0	31.9	1.034
hsa-miR-148b*	36.0	36.0	1.033
hsa-miR-340	36.0	36.0	1.031
hsa-miR-518f	31.0	30.9	1.029
hsa-miR-224	33.0	33.0	1.025
hsa-miR-26a-1*	33.0	33.0	1.021
hsa-miR-30a-5p	28.0	27.9	1.015
hsa-miR-197	26.1	26.0	1.011
hsa-miR-195	30.0	30.0	1.009
hsa-miR-144*	34.0	34.0	1.005
hsa-miR-335	31.9	31.9	1.003
hsa-miR-744	31.0	31.0	0.998
hsa-miR-516-3p	37.0	37.0	0.989
hsa-miR-629	34.0	34.0	0.986

hsa-miR-320b	31.0	31.0	0.986
hsa-miR-30e-3p	30.9	31.0	0.984
hsa-miR-652	35.0	35.0	0.980
hsa-miR-30d	30.9	31.0	0.975
hsa-miR-27b	31.9	31.9	0.972
hsa-miR-19b	26.0	26.1	0.969
hsa-miR-885-5p	27.9	28.0	0.968
hsa-miR-374b	30.9	31.0	0.961
hsa-miR-942	33.7	33.8	0.959
hsa-miR-377	23.9	24.0	0.954
hsa-miR-301a	32.9	33.0	0.950
hsa-miR-495	32.9	33.0	0.950
hsa-miR-139-5p	28.9	29.0	0.945
hsa-miR-532-5p	35.9	36.0	0.941
hsa-miR-93	28.9	29.0	0.920
hsa-miR-18a	34.0	34.2	0.916
hsa-miR-532-3p	33.8	34.1	0.840
hsa-miR-193b	29.8	30.2	0.787
hsa-miR-221	28.4	29.0	0.666
hsa-miR-660	31.2	31.9	0.602
hsa-miR-125a-5p	35.1	35.9	0.578
hsa-miR-636	32.1	32.9	0.571
hsa-miR-92a	25.2	26.0	0.568
hsa-miR-143	33.0	33.9	0.566
hsa-miR-132	35.1	36.0	0.543
hsa-miR-126	26.1	27.0	0.535
hsa-miR-185	31.0	32.0	0.534
hsa-miR-127-3p	34.0	35.0	0.517
hsa-miR-27a	32.0	33.0	0.513
hsa-miR-99b	32.0	33.0	0.512
hsa-miR-671-3p	33.0	34.0	0.507
hsa-miR-1255b	33.0	34.0	0.503
hsa-miR-374a	31.9	32.9	0.501
hsa-miR-328	29.0	30.0	0.500
hsa-miR-191*	34.0	35.0	0.498
hsa-miR-324-5p	33.0	34.0	0.498
hsa-miR-539	34.0	35.0	0.497
hsa-miR-151-5p	32.0	33.0	0.497
hsa-miR-30c	27.9	28.9	0.496
hsa-miR-1233	26.0	27.0	0.495
hsa-miR-26b	31.0	32.0	0.493
hsa-miR-451	27.9	29.0	0.491
hsa-miR-629	34.0	35.0	0.485
hsa-miR-28-5p	33.0	34.0	0.473

hsa-miR-34b	30.9	32.2	0.432
hsa-miR-485-3p	31.2	32.9	0.295
hsa-miR-181a	30.2	32.0	0.294
hsa-miR-193a-5p	30.0	31.8	0.284
hsa-miR-125b	33.0	34.9	0.272
hsa-miR-379	34.1	36.0	0.270
hsa-miR-433	31.0	33.0	0.263
hsa-miR-30b	28.0	30.0	0.258
hsa-miR-203	34.0	36.0	0.255
hsa-miR-15b	32.0	34.0	0.254
hsa-miR-1274b	21.0	23.0	0.253
hsa-miR-375	31.0	33.0	0.251
hsa-let-7e	26.9	28.9	0.250
hsa-miR-886-3p	33.0	35.0	0.246
hsa-miR-1274a	26.0	28.0	0.245
hsa-miR-27a*	34.0	36.0	0.245
hsa-miR-130b	31.0	33.0	0.242
hsa-miR-148a	32.0	34.0	0.240
hsa-let-7d	29.0	31.0	0.240
hsa-miR-101	33.9	36.0	0.235
hsa-miR-122	29.1	31.8	0.155
hsa-miR-130a	31.0	34.0	0.123
hsa-miR-1285	29.0	32.0	0.120
hsa-miR-142-3p	28.9	32.0	0.120
hsa-miR-338-5p	32.0	35.1	0.116
hsa-miR-1303	29.3	33.2	0.070
hsa-miR-515-3p	20.2	24.1	0.067
hsa-miR-381	20.0	25.1	0.028
hsa-miR-302c	15.4	22.0	0.010

HPT: hypertension; AAAD: acute Stanford type A aortic dissection.

Supplementary Table 3. Differentially expressed serum miRNAs using TLDA analysis.

miRNAs	Fold change		
	HPT+/AAAD+ vs. AAAD-	HPT-/AAAD+ vs. AAAD-	HPT+/AAAD+ vs. HPT+/AAAD-
hsa-miR-432	30.9	4.0	63.0
hsa-miR-1290	23.7	5.9	16.4
hsa-miR-186	23.5	2.8	8.4
hsa-miR-191	16.9	3.9	16.9
has-miR-155	7.7	2.0	3.7
hsa-miR-25	2.9	3.8	4.2
hsa-miR-29a	2.1	--	2.0
hsa-miR-185	0.5	0.0	0.5
hsa-miR-126	0.5	0.0	0.5
hsa-miR-27a	0.5	0.3	0.5
hsa-miR-26b	0.5	0.1	0.5
hsa-miR-30c	0.5	0.0	0.5
hsa-miR-28-5p	0.5	0.3	0.5
hsa-let-7e	0.4	0.0	0.2
hsa-miR-451	0.3	0.0	0.5
hsa-miR-1274a	0.3	0.0	0.2
hsa-let-7d	0.3	0.1	0.2
hsa-miR-1274b	0.3	0.0	0.3
hsa-miR-130b	0.2	0.0	0.2
hsa-miR-515-3p	0.1	0.1	0.1
hsa-miR-381	0.0	0.0	0.0
hsa-miR-302c	0.0	0.0	0.0

HPT: hypertension; AAAD: acute Stanford type A aortic dissection.

Supplementary Table 4. Differentially expressed serum miRNAs determined by RT-qPCR*.

miRNAs	Fold change		*P*-value		Result
	AAAD+ vs. AAAD-	HPT+/AAAD+ vs. HPT+/AAAD-	AAAD+ vs. AAAD-	HPT+/AAAD+ vs. HPT+/AAAD-	
hsa-miR-25	3.326±0.414	5.237±0.670	<0.001	<0.001	significant
hsa-miR-29a	2.850±0.042	3.022±0.509	<0.001	0.001	significant
has-miR-155	2.583±0.258	2.227±0.266	<0.001	<0.001	significant
hsa-miR-26b	0.351±0.047	0.306±0.055	<0.001	<0.001	significant
hsa-miR-27a	2.740±0.729	1.101±0.351	0.012	0.785	non-significant
hsa-miR-1290	1.371±0.309	1.912±0.646	0.459	0.191	non-significant
hsa-miR-191	1.344±0.206	1.605±0.320	0.121	0.097	non-significant
hsa-miR-451	1.211±0.178	0.825±0.126	0.344	0.299	non-significant
hsa-miR-186	1.208±0.170	1.084±0180	0.277	0.698	non-significant
hsa-miR-432	1.170±0.306	1.076±0.409	0.570	0.854	non-significant
hsa-miR-185	0.853±0.172	0.707±0.131	0.506	0.176	non-significant
hsa-miR-30c	0.756±0.284	0.799±0.339	0.394	0.594	non-significant
hsa-miR-28-5p	0.524±0.165	0.635±0.102	0.002	0.082	non-significant
hsa-miR-126	0.457±0.190	0.543±0.251	0.006	0.089	non-significant
hsa-let-7d					Detection rate <75%
hsa-let-7e					Detection rate <75%
hsa-miR-1274a					Detection rate <75%
hsa-miR-1274b					Detection rate <75%
hsa-miR-130b					Detection rate <75%
hsa-miR-302c					Detection rate <75%
hsa-miR-381					Detection rate <75%
hsa-miR-515-3p					Detection rate <75%

HPT: hypertension; AAAD: acute Stanford type A aortic dissection.

*The fold changes of miRNAs are presented as mean ± SEM.

Statistical comparison was performed by using Student's t-test.

Supplementary Table 5. Receiver operating characteristic curves in the training set and validation set.

Groups	Risk score	AUC (95% CI)	P-value	Cutoff point	Sensitivity	Specificity	PV+	PV-
Training set AAAD+ vs. AAAD-	panel	0.995(0.982-1.000)	<0.001	46.50%	96.00%	100.00%	100.00%	96.77%
	miR-25	0.881(0.787-0.976)	<0.001	1.264	92.00%	76.67%	76.67%	92.00%
	miR-29a	0.899(0.805-0.992)	<0.001	1.488	80.00%	93.33%	90.91%	84.85%
	miR-155	0.863(0.762-0.963)	<0.001	1.464	84.00%	83.33%	80.77%	86.21%
	miR-26b	0.911(0.829-0.992)	<0.001	0.533	88.00%	90.00%	88.00%	90.00%
Validation set AAAD+ vs. AAAD-	panel	0.978(0.957-0.999)	<0.001	46.50%	89.06%	94.83%	95.00%	88.71%
	miR-25	0.857(0.791-0.922)	<0.001	1.264	81.25%	74.14%	77.61%	78.18%
	miR-29a	0.897(0.840-0.955)	<0.001	1.488	78.13%	86.21%	86.21%	78.13%
	miR-155	0.871(0.805-0.937)	<0.001	1.464	84.38%	77.59%	80.60%	81.82%
	miR-26b	0.803(0.723-0.883)	<0.001	0.533	65.63%	82.76%	80.77%	68.57%
Training set HPT+/AAAD+ vs. HPT+/AAAD-	panel	0.996(0.981-1.000)	<0.001	36.89%	100.00%	93.33%	93.75%	100.00%
	miR-25	0.929(0.822-1.000)	<0.001	1.353	93.33%	86.67%	87.50%	92.86%
	miR-29a	0.916(0.798-1.000)	<0.001	1.354	86.67%	93.33%	92.86%	87.50%
	miR-155	0.836(0.680-0.991)	0.002	1.457	80.00%	86.67%	85.71%	81.25%
	miR-26b	0.924(0.819-1.000)	<0.001	0.500	86.67%	93.33%	92.86%	87.50%
Validation set HPT+/AAAD+ vs. HPT+/AAAD-	panel	0.985(0.965-1.000)	<0.001	36.89%	97.73%	82.76%	89.58%	96.00%
	miR-25	0.920(0.859-0.981)	<0.001	1.353	88.64%	86.21%	90.70%	83.33%
	miR-29a	0.906(0.834-0.978)	<0.001	1.354	88.64%	75.86%	84.78%	81.48%
	miR-155	0.850(0.758-0.942)	<0.001	1.457	77.27%	82.76%	87.18%	70.59%
	miR-26b	0.810(0.708-0.913)	<0.001	0.500	65.91%	89.66%	90.63%	63.41%

AUC, area under curves; CI, confidence interval; PV+, positive predictive value; PV-, negative predictive value. HPT: hypertension; AAAD: acute Stanford type A aortic dissection.

.

Supplementary table 6. Demographic and clinical features of HPT+/AAAD+ patients compared with HPT+/AAAD- individuals in blinded trail set.

	Blinded trail (n=30)		
	HPT+/AAAD+ (n=15)	HPT+/AAAD- (n=15)	P-value (HPT+/AAAD+ vs. HPT+/AAAD)
Age (years)	59.4±9.83	54.07±7.04	0.099[a]
Female	6	4	0.439[b]
Smoker	3	0	0.224[b]
Alcohol abuse	4	0	0.100[b]
Hypertension	15	15	--
Hyperlipidemia	2	1	0.543[b]
Atherosclerosis	2	0	0.483[b]
Aortic aneurysm	0	0	--
Marfan's syndrome	0	0	--
Bicuspid aortic valve	0	0	--
Cocaine abuse	0	0	--
Diabetes	3	2	0.624[b]
Previous AD	2	0	0.483[b]

HPT: hypertension; AAAD: acute Stanford type A aortic dissection.

Statistical comparison was performed by using Student's t-test (a) or two-sided λ^2 test (b).

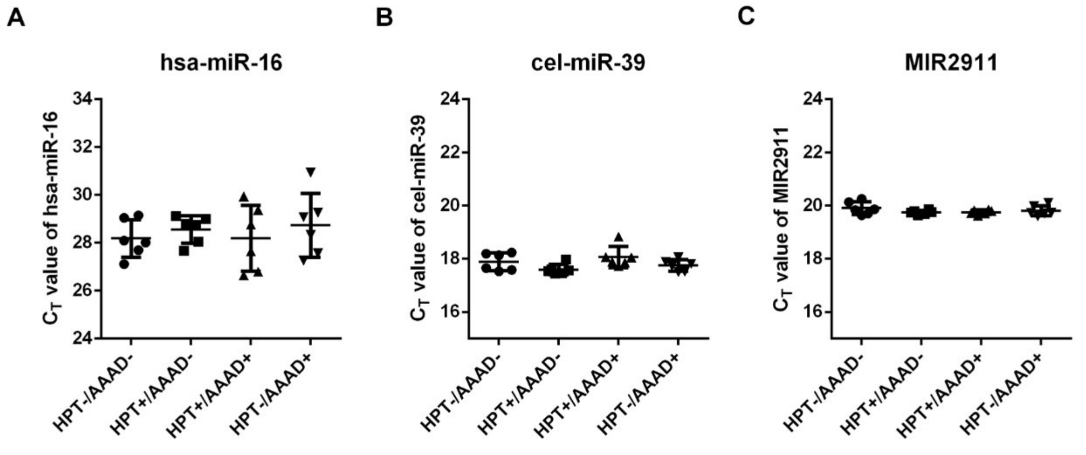

Supplementary Figure 1. Expression levels **(**C_T value) of endogenous and exogenous miRNAs. 100 μL of blood serum sample was mixed with 1 mL of TRIzol Reagent, 5 μL of synthetic MIR2911 or Cel-miR-39 were added into the mixture as external controls. (A). The expression level of endogenous has-miR-16 in different group of serum samples. (B) and (C) The expression levels of exogenous cel-miR-39 and MIR2911 in serum samples as external controls. HPT: hypertension; AAAD: acute Stanford type A aortic dissection.
